# Phytochemical Analysis, Antioxidant Activity and Bioassay-Guided Isolation of Acetylcholinesterase and Butyrylcholinesterase Inhibitors from *Horsfieldia polyspherula* Bark (Myristicaceae)

**DOI:** 10.21315/tlsr2024.35.3.8

**Published:** 2024-10-07

**Authors:** Mohammed Idris, Mohamad Nurul Azmi, Thaigarajan Parmusivam, Unang Supratman, Marc Litaudon, Khalijah Awang

**Affiliations:** 1 Natural Products and Synthesis Organic Research Laboratory, School of Chemical Sciences, Universiti Sains Malaysia, 11800 USM Pulau Pinang, Malaysia; 2 Department of Chemistry, Federal University, Yusufari Road, Gashua, 671106 Yobe, Nigeria; 3 School of Pharmacy, Universiti Sains Malaysia, 11800 USM Pulau Pinang, Malaysia; 4 Department of Chemistry, Faculty of Mathematics and Natural Sciences, Universitas Padjadjaran, 45363 Jatinangor, Indonesia; 5 Institut de Chimie des Substances Naturelles, CNRS-ICSN UPR 01, Universite Paris-Sud 11, Av. de la Terrasse, 91198 Gif-sur-Yvette, France; 6 Department of Chemistry, Faculty of Science, Universiti Malaya, 50603 Kuala Lumpur, Malaysia

**Keywords:** Myristicaceae, *Horsfieldia polyspherula*, Alzheimer’s disease, Acetylcholinesterase, Butyrylcholinesterase

## Abstract

Alzheimer’s disease (AD) is a neurodegenerative condition brought on by aging and characterised by progressive decline in cognitive function and abnormalities in the central cholnergic system. *β*-amyloid deposits, neurofibril tangle aggregation, oxidative stress or reduced level of acetylcholine are a few causes that have been linked to AD. In this study, the bioassay-guided isolation from ethyl acetate (EtOAc) extract of *Horsfieldia polyspherula* bark led to the isolation of nine compounds namely, 16-phenylhexadecanoic acid (**1**), undecylbenzene (**2**), 3,4-dihydroxybenzoic acid (**3**), dodecanoic acid (**4**), tetradecanoic acid (**5**), pentadecanoic acid (**6**), 1-tridecene (**7**), stigmasterol (**8**) and trimyristin (**9**). Phytochemical analysis revealed the presence of flavonoids, steroids, lignin, alkaloids, phytosterol and triterpenoids. The DPPH scavenging activity of EtOAc extract was related to the phenolic content (116.67 ± 16.98 GAE mg/g) and other non-phenolics such as lower fatty acids. Meanwhile, the DPPH scavenging activity was found to be concentration-dependent and correlated with both flavonoid and phenolic content. Furthermore, EtOAc and methanol (MeOH) extracts of *H. polyspherula* bark showed significant inhibitory activity at 100 μg/mL on acetylcholinesterase (AChE) and butyrylcholinesterase (BuChE), with EtOAc extract showing 77.2% and 64.1% inhibition and MeOH extract showing 37.5% and 39.2% inhibition, respectively. Additionally, the IC_50_ for BuChE and AChE of the EtOAc extract were found to be effective, with 15.41 ± 0.78 μg/mL and 7.67 ± 0.13 μg/mL, respectively. Compound **1** exhibited dual inhibition of 40.99 ± 1.99 μM (BuChE) and 46.83 ± 2.44 μM (AChE), while compounds **2** and **3** showed IC_50_ values above 200 μM. This study revealed that this plant shows a significant potential as anti-cholinesterase focusing on acetylcholinesterase (AchE) and butyrylcholinesterase (BuChE). This is the first report on *Horsfieldia polyspherula* and their biological activity.

**Highlights**
- The bioassay-guided isolation from Malaysian *Horsfieldia polyspherula* bark (Myristicaceae) led to the isolation of 9 compounds.
- The IC_50_ for butyrylcholinesterase (BuChE) and acetylcholinesterase(AChE) of the EtOAc extract were found to be effective, with 15.41 ±0.78 μg/mL and 7.67 ± 0.13 μg/mL, respectively.
- Compound **1** exhibited dual inhibition with IC_50_, 40.99 ± 1.99 μM for BuChE and 46.83 ± 2.44 μM for AChE.
- This is the first report on *Horsfieldia polyspherula* and their biological activity as cholinesterase inhibitors.

## INTRODUCTION

Alzheimer’s disease (AD) was first reported by Alois Alzheimer, a German scientist, in 1907 as an age-related neurodegenerative process characterised by a progressive loss of cognitive abilities closely associated with a defect in the central cholinergic system. Although its aetiology is still poorly understood, several factors, such as beta-amyloid deposits, neurofibril tangle aggregation, oxidative stress, and low levels of acetylcholine, are thought to play significant roles in the pathology of the disease (Anwal 2021). The National Health and Morbidity Survey reported that 8% of Malaysians suffered from different degrees of dementia in 2008. This coincides with the Alzheimer’s Disease Foundation in Malaysia, which estimated that 260,000 (11%) adults were suffering from dementia and projected that this figure would rise to 800,000 (312%) by 2050. Globally, dementia was reported to have affected 46 million people and is expected to increase to 131 million by the year 2050 unless a cure or treatment for its onset or progression is found (Lai *et al*., 2022; Alzheimer’s Association Report 2023). The pharmacological treatment of AD is primarily based on the use of cholinesterase inhibitors and treating oxidative stress can increase neuroprotective survival (Pourparizi *et al*. 2023). Oxidative stress refers to an imbalance between the systemic manifestation of reactive oxygen species (ROS) and the ability of the biological system to efficiently detoxify these reactive intermediates and facilitate the repair of the resulting damage. This imbalance plays a role in several diseases, including ageing and AD (Aran & Singh 2023). ROS are produced endogenously during respiration in the mitochondria while externally are produced by various pollutants such as smoke, radiation, drugs and temperature (Widowati *et al*. 2020). This increased radiolysis can lead to cellular senescence and apoptosis, contributing to the development of diseases such as ageing and Alzheimer’s (Beura *et al*. 2022). Antioxidants are used to mitigate these adverse effects. Antioxidants are molecules that can donate electrons to free radicals without affecting their stability (Jiangseubchatveera *et al*. 2023). This process stabilises the free radicals, reduces their reactivity and thus prevents the progression of chronic diseases (Megawati *et al*. 2023). Both extracts and compounds isolated from *H. macrobotrys, H. motley* and *H. spicata* were reported to have exhibited various degrees of scavenging activity against DPPH and 2,2′-azino-bis(3-ethylbenzothiazoline-6-sulfonic acid) (ABTS) (Ramadhan & Phuwapraisirisan 2015; Ramadhan *et al*. 2018; Megawati *et al*. 2023). Cholinesterase (ChE) inhibitors are commonly prescribed to enhance cholinergic signalling in the hopes of improving cognitive function, which is partly compromised by the degeneration of cholinergic innervation in the hippocampus and cortex. The search for effective treatments for AD has been ongoing for decades because current drugs not only impact the cholinergic pathway but also affect the immune system (Pohanka 2014). Acetylcholinesterase and butyrylcholinesterase are the main agents in the hydrolysis of acetylcholine. It was reported that acetylcholinesterase levels decrease in patients with AD while butyrylcholinesterase increases.

Natural products played a vital role in drug discovery, hence the need to search for new inhibitors from *Horsfieldia* species. As the *Myristicaceas* family was reported to possess some acetylcholinesterase inhibitory activity (Al-Mekhlafi *et al*. 2013), preliminary investigation by our group reveals the potential of *Horsfieldia polyspherula* as a cholinergic inhibitor. The genus *Horsfieldia* (Myristicaceae) consists of more than a hundred species distributed across Malaysia, Sri Lanka, the Caroline Islands, Northeast India and Southeast China. It is known for its spices and is referred to as nutmeg in English, “*penarahan*” or “*darahdarah*” in Malay, and “*jela-bala*” in Kenya (Al-Mekhlafi *et al*. 2013). They are dioecious trees that have ridged twigs usually several times but not from the bottom and alternate (distichous) rows of leaves about the twigs. They have up to 45 cm long leaves blade that is breakable when dried. The flowers are usually small ranging from 0.5 mm–4 mm, where their male bud is smaller than that of the female bud (Armstrong & Wilson 1978). This genus is an important member of Myristicaceae used as a folk remedy to treat diarrhoea, intestinal disorder, sore throat, stomach ache and pimples (Du *et al*. 2017). Extracts from the leaves, stem and root bark of *H.helwigii* have been reported to possess antibacterial and protozoan activities. Furthermore, the screening of the dichloromethane (DCM) extract from the stem, leaves and roots of *H. polyspherula* has shown a 90% inhibitory activity against *α*-glucosidase and *α*-amylase. Additionally, EtOAc and chloroform extracts from *H.superba* have been reported to exhibit more than 70% inhibitory potency against AChE (Jemain *et al*. 2011; Al-Mekhlafi *et al*. 2013). Previous phytochemical investigation on this genus led to the isolation of alkaloids, lignans, terpenoids, essential oils, arylalkanones, flavones, steroids and fatty acids (Ramadhan &Phuwapraisirisan 2015). The bioassay-guided fractionation procedure is commonly employed in drug discovery research due to its effectiveness in directly linking the analysed extract and targeted compounds through bioassys carried out at each stage of the fractionantion (Tillekeratne *et al*. 1982).

Based on preliminary work and reports from our group on the *Horsfieldia* genus, this research aim to search for phytoconstituents present in EtOAc extract of *H. polyspherula* bark as well as test for their inhibitory activity against AChE and BuChE in order to provide further scientific information on the application of *H.polyspherula*.

## MATERIALS AND METHOD

### General

Solvents and reagents used for this research were sourced from Merck (Germany), Sigma-Aldrich, Friedmann Schmidt Chemical, Fluka Chemie and Qrec (Asia). Flash chromatographic techniques under control pressure were used with silica gel 60 (0.06 mm–0.2 mm) and subsequent purification with 230–400 and 70–230 silica gel mesh, pre-coated TLC silica 60 gel F_234_ 20 cm × 20 cm on aluminium sheet, Merck were used, 254 nm UV lamp, Vanillin-sulfuric acid reagent and heating gun were used for visualisation. Fourier transform infra-red spectra were recorded on PerkinElmer FT-IR Spectrometer (Frontier), applying guage between 100 to 119 followed by scanning the spectra between 600 cm^−1^–4000 cm^−1^ and subsequent smoothing and peak picking, while the oily sample was applied directly onto the diamond face after cleaning the diamond face with the appropriate solvent and background scan. The sample results were recorded without tying the gauge. NMR spectra were recorded using Bruker AvanceIII 500 spectrometer operated at 500 MHz for 1D and 2D NMR experiments (^1^H-^1^H COSY, HSQC and HMBC) and 125 MHz for ^13^C-NMR and DEPT135 experiments. The samples were dissolved in an appropriate solvent of either chloroform-D_1_ (^1^H: 7.26 ppm, ^13^C: 77.0 ppm; Merck) or methanol-D_4_ (^1^H: 3.31, 4.78 ppm, ^13^C: 49.2 ppm; Merck) and TMS as an internal standard. High-resolution electron ionisation mass spectrometry (HRESIMS) was performed using Time-of-Flight Mass Spectrometer (TOF-MS) (Waters®, Massachusetts, United States). Shimadzu UV-2600 UV-Vis Spectrophotometer and Spectrophotometric microplate reader (Bostch Biotek) were both used for the UV-Vis spectrum.

### Plant Material

The dried bark of *H. polyspherula* J. Sinclair was collected from Felda Kertih, Ulu Kertih, Terengganu, identified by Teo L. E., a botanist at Universiti Malaya. The voucher specimen (KL4620) has been deposited at the herbarium of the Department of Chemistry, Faculty of Science, Universiti Malaya, Kuala Lumpur, Malaysia.

### Extraction and Isolation of Compounds

The air-dried bark of *H. polyspherula* (1 kg) was macerated with EtOAc (3 L) at room temperature for 72 h and filtered. The solvent was evaporated under reduced pressure at 40°C. This process was repeated thrice and yielded 21.0 g (2.1% yield) of extract. Upon successful completion of EtOAc extraction, methanol was used as a solvent and followed the same maceration technique as reported for EtOAc and yielded 47.0 g (4.7% yield). Both extracts were submitted for butyrylcholinesterse and acetylcholinesterase assays.

The EtOAc extract (20.0 g) was subjected to flash column chromatography (CC) over silica gel using a gradient solvent of *n*-hexane/EtOAc and dichloromethane (DCM)/MeOH (0 → 100 and 80 → 20; v/v) to yield 11 fractions (F1–F11). Fraction 4 (1.9 g) was fractionated using column chromatography (CC) over silica gel with *n*-hexane and EtOAc gradient system (100:0 → 70:30; v/v) to obtain three fractions (F4.1–F4.3). Fraction F4.2 was pure Compound **6** (30 mg), while fraction F4.3 (200 mg) was subjected to a second CC by using the same solvent system on CC to give Compounds **6** (10 mg) and **5** (4 mg), respectively.

Further purification of F4.1 were found to be Compounds **6** (7 mg) and **8** (5 mg),respectively. Fraction F5 was subjected to CC over silica gel, using *n*-hexane/EtOAc (100:0 → 55:45 v/v) as eluents to give three sub-fractions, i.e, F5.1–F5.3. Compound **4** (3 mg) was obtained from F5.2. Fraction F7 was further subjected to CC over silica gel with *n-*hexane/EtOAc (100:0 → 55:45; v/v) as eluent yielding three fractions after pooling (F7.1–F7.3), where F7.2 was found to be compound **3** (500 mg). Finally, Fraction F9 afforded Compounds **7** (3 mg), **9** (10 mg) and **2** (13.5 mg), while fraction F10 yielded Compound **1** (11 mg). The structure of isolated compounds were determined by spectroscopic analysis and comparism with published data.

16-phenylhexadecanoic acid (**1**):

Dark brown gummy, yield: 0.011 g (0.0011 %); M.p.: 78°C–80°C; FT-IR(ATR) V_max_ cm^−1^: 3414 (O-H), 2928 (Csp^3^-H), 2852 (Csp-H), 1708 (C=O).1463 (Csp^3^-H, Csp^3^-C), 753 (Csp^2^-C); HRESIMS (^−^ESI) [M-H]^−^: 331.2625,cal.: 331.2637, correspond to C_22_H_35_O_2_; ^1^H-NMR (500 MHz, CDCI_3_) δ_H_ 7.21(2H, t, *J* = 7.5 Hz, H-3′, H-5′). 7.11 (2H, d. *J* = 6.0 Hz, H-2′, H-6′), 7.10 (1H,t, *J* = 2.0 Hz, H-4′), 2.53 (2H, t, *J* = 8.0 Hz, H-16), 2.29 (2H, dt, *J* =7.5 Hz,*J* = 5.0 Hz, H-2), 1.62-1.52 (4H, m, H-3, H-15), 1.19 (22H, br s, H-4–H-14).^13^C-NMR (125 MHz CDCl_3_), δ_C_: 178.0 (C-1), 141.8 (C-1′), 128.6 (C-2′, C-6′),128.4 (C-3′ C-5′), 125.8 (C-4′), 36.1 (C-16), 33.9 (C-2), 32.1 (C-15), 29.9-29.3 (overlapping signal C-4-C-14), 24.9 (C-3) (Supplementary Material S1–S2) (Gonfa *et al*. 2023).

Undecylbenzene (**2**):

Light yellow oil, yield: 0.0135 g (0.00135 %): FT-IR (ATR) V_max_ cm^−1^: 2914 (Csp^3^-H), 2847 (Csp^3^-H), 1461 (Csp^3^-H, Csp^2^-C), 723 (Csp^3^-H, Csp^2^-C); HRESIMS (^+^ESI) [M+Na]^+^: 255.2089, cal.: 255.2089 correspond to C_17_H_28_Na; ^1^H-NMR (500 MHz. CDCl_3_) δ_H_ 7.24.2 (2H, t, *J* = 7.5 Hz, H-3′, H-5′), 7.13 (2H, d, *J* = 5.5 Hz, H-2′, H-6′), 7.11 (1H, t, *J* = 2.5 Hz, H-4′). 2.54 (2H, t, *J* = 7.5 Hz, H-1), 2.29 (2H, dt, *J* = 7.5 Hz, 2.5, H-2), 1.30-1.16 (16H, m, H-3–H-10), 0.83 (3H, t, *J* = 7.5 Hz, H-11), ^13^C-NMR (125 MHz CDCl_3_), δ_C_ 142.9 (C-1′),128.4 (C-2′, C-6′), 128.2 (C-3′, C-5′), 125.5 (C-4′), 35.9 (C-1), 33.5 (C-2), 31.5 (C-8), 29.7-29.0 (overlapping signal C-3-C-7), 24.7 (C-9), 22.7 (C-10), 14.1 (C-11) (Supplementary Material S3–S4) (Sardar *et al*. 2022).

3,4-dihydroxybenzoic acid (**3**):

Light brown granule, yield: 0.5 g (0.05 %); M.p.: 201°C–203°C; FT-IR (ATR) V_max_ cm^−1^: 3181 (O-H), 1664 (C=O), 1598 (Csp^2^-C), 1290 (C-O), 933 (O H), 760 (Csp^2^-C); HRESIMS (^+^ESI) [M+Na]^+^: 177.0156, cal.: 177.0164, correspond to C_7_ H_6_O_4_Na; ^1^H-NMR (500 MHz, CD_3_OD): δ_H_ 7.44 (1H, d, *J* = 1.5 Hz, H-2), 7.42 (1H, d, *J* = 2.0 Hz, H-6), 6.8 (1H, d, *J* = 8.0 Hz, H-5); ^13^C-NMR (125 MHz, CD_3_OD): δ_C_ 170.2 (C-7), 151.5 (C-4), 146.1 (C-3), 123.9 (C 6), 123.2 (C-1), 117.8 (C-2), 115.8 (C-5) (Supplementary Material S5–S6) (Ourhzif *et al*., 2022).

Dodecanoic acid (Lauric acid) (**4**):

White amorphous, yield: 0.003 g (0.0003 %); M.p.: 46°C–48°C; FT-IR (ATR) V_max_ cm^−1^: 3360 (O-H), 2938 (Csp^3^-H), 2857 (Csp^3^-H), 1741 (C=O), 1214 (C-O), 748 (Csp^3^-H); HRESIMS (^+^ESI) [M+H]^+^: 201.1886, cal.: 201.2307, correspond to C_12_H_25_O_2_; ^1^H-NMR (500 MHz, CDCl_3_) δ_H_ 2.36 (2H, t, *J* = 7.5 Hz, H-2), 1.62 (2H, m, H-3), 1.25 (16H, br s, H-4–H-11), 0.88 (3H, t, *J* = 7.0 Hz, H-12), ^13^C-NMR (125 MHz CDCl_3_), δ_C_ 177.5 (C-1), 33.8 (C-2), 32.1 (C-3), 29.9–29.3 (overlapping signal C-4–C-9), 24.9 (C-10), 22.9 (C-11), 14.3 (C-12) (Supplementary Material S7–S8) (Batsika *et al*. 2022).

Tetradecanoic acid (myristic acid) (**5**):

White amorphous, yield: 0.004 g (0.0004 %); M.p: 54°C–57°C; FT-IR (ATR) V_max_ cm^−1^: 2912 (Csp^3^-H), 2852 (Csp^3^-H), 1703 (C=O), 1460 (Csp^3^-H). 1243 (C-O), 893 (O-H), 718 (Csp^3^-H); HRESIMS (^+^ESI) [M+2H]^+^: 230.2960, cal.: 230.2246, correspond to C_14_H_30_O_2_; ^1^H-NMR (500 MHz, CDCI_3_): δ_H_ 2.30 (2H, t, *J* = 7.5 Hz, H-2), 1.60 (2H, m. H-3), 1.19 (20H, br s, H-4–H-13), 0.88 (3H, t, *J* = 7.0 Hz, H-14), ^13^C-NMR (125 MHz CDCl_3_) δ_C_ 178.0 (C-1), 34.0 (C-2), 32.1 (C-12), 29.9–29.3 (overlapping signal C-4–C-11), 24.9 (C-3), 22.9 (C-13), 14.3 (C-14) (Supplementary Material S9–S10) (Yang *et al*. 2021).

Pentadecanoic acid (**6**):

White cake-like amorphous, yield: 0.047 g (0.0047 %); M.p.: 78°C–80°C; FT-IR (ATR) V_max_ cm^−1^: 2915 (Csp^3^-H), 2847 (Csp^3^-H), 1701 (C=O), 1462 (Csp^3^-H). 1304 (C-O), 914 (O-H), 724 (Csp^3^-H); HRESIMS (^+^ESI) [M+Na]^+^: 265.2557, cal.: 265.2124, correspond to C_14_H_30_O_2_Na; ^1^H-NMR (500 MHz, CDCI_3_) δ_H_ 2.28 (2H, t, *J* = 7.5 Hz, H-2), 1.56 (2H, m, H-3), 1.19 (22H, br s, H-4–H-14), 0.82 (3H, t. *J* = 7.0 Hz, H-15), ^13^C-NMR (125 MHz CDCl_3_), δ_C_ 178.6 (C-1), 33.9 (C-2), 32.1 (C-13). 29.9–29.2 (overlapping signal C-4–C-12), 24.9 (C-3), 22.9 (C-14), 14.3 (C-15) (Supplementary MaterialS11–S12) (Kumar *et al*. 2023).

1-tridecene (**7**):

Colourless oil, yield: 0.003 g (0.0003%); FT-IR (ATR) V_max_ cm^−1^: 2916(Csp^3^-H), 2849 (Csp^3^-H), 1463 (Csp^3^-H, Csp^2^-H), 906 (Csp^2^-C), 720(Csp^3^-H); HRESIMS (^+^ESI) [M+H]^+^: 183.2129, cal.: 183.2113, correspond to C_13_H_27_; _1_H-NMR (500 MHz. CDCl_3_) δ_H_ 5.81 (1H, dtd, *J* = 17.0 Hz, *J* = 10.5 Hz, H-2), 5.00 (1H, dd, *J* = 17.0 Hz, *J* = 2.0 Hz, H-1α), 4.93 (1H, td, *J* = 10.2 Hz,*J* = 1.0 Hz, H-1β), 2.06-1.99 (2H, m, H-3), 1.25 (18H, br s, H-4–H-12), 0.88(3H, t, *J* = 7.0 Hz, H-13), ^13^C-NMR (125 MHz CDCl_3_), δ_C_ 139.3 (C-2), 114.1(C-1), 33.8 (C-3), 32.0 (C-11), 29.7–29.2 (overlapping signal C-4–C-10),22.7 (C-12), 14.1 (C-13) (Supplementary Material S13–S14) (Lei & Rovis 2019).

Stigmasterol (**8**):

White crystal, yield: 0.005 g (0.0005%); M.p.: 147°C–148°C: FT-IR (ATR) V_max_ cm^−1^: 3414 (O-H), 2937 (Csp^3^-H), 2862 (Csp^3^-H), 1465 (Csp^3^-H, Csp^2^-H), 1374 (Csp^3^-H), 1044 (C-O); HRESIMS (^+^ESI) [M+H]^+^: 413.3755, cal.: 413.3788, correspond to C_29_H_49_O; ^1^H-NMR (500 MHz, CDCI_3_): δ_H_ 5.33 (1H, d, *J* = 5.5 Hz, H-6), 5.13 (1H, dd, *J* = 8.5 Hz, *J* = 15 Hz, H-22), 4.99 (1H, dd, *J* = 8.5 Hz, *J* = 15 Hz, H-23), 3.55 (3H, m, H-3), 2.00 (1H, m, H-20), 1.27 (2H, s. H-21), 0.90 (3H, t, *J* = 7.4 Hz, H-29), 0.84 (3H, s, H-27), 0.70 (3H, s, H-19); ^13^C-NMR (125 MHz, CDCI_3_): δ_C_ 140.8 (C-5), 138.3 (C-22), 129.3 (C-23), 121.7 (C-6), 71.8 (C-3), 56.8 (C-14), 56.1 (C-17), 50.1 (C-9), 45.8 (C-13), 42.3 (C-4), 39.8 (C-12), 37.3 (C-1), 36.5 (C-10), 36.2 (C-20), 33.9 (C-8), 31.9 (C-7), 31.7 (C-2), 29.1 (C-25), 28.3 (C-16), 26.1 (C-24), 24.3 (C-15), 23.1 (C-21), 21.1 (C-11), 19.8 (C-26), 19.4 (C 19), 19.0 (C-27), 18.8 (C-28), 12.0 (C-29), 11.9 (C-18) (Supplementary Material S15–S16)(Kurniasih *et al*. 2021).

Trimyristin (**9**):

White greasy solid, yield: 0.010 g (0.0010%); M.p.; 50°C–51°C; FT-IR (ATR) V_max_ cm^−1^: 2918 (Csp^3^-H), 2854 (Csp^3^-H), 1730 (C=O), 1471 (Csp^3^-H), 1168 (C-O). 721 (Csp^3^-H); HRESIMS (^+^ESI) [M]^+^: 722.6813, cal.: 722.6424, correspond to C_45_H_86_O_6_; ^1^H-NMR (500 MHz, CDCl_3_) δ_H_ 5.19 (1H, m, H-2), 4.22 (2H, dd, *J* = 12.0 Hz, *J* = 4.5 Hz, H-1α, H-3α), 4.06 (2H, q, *J* = 12.0 Hz, *J* = 6.0 Hz, H-1β, H-3β), 2.23 (6H, dt, *J* = 7.5 Hz, *J* = 2.5 Hz, H-2′, H-2″, H-2‴), 1.53 (6H, m. H-3″, H-3″, H-3‴), 1.18 (160H, br s, H-4′, H-4″,H-4‴ - H-13′, H-13″, H-13‴), 0.80 (9H, t, *J* =7.5 Hz, H-14′, H-14″, H-14‴);^13^C-NMR (125 MHz CDCl_3_), δ_C_ 173.4 (C-1″, C-1‴), 173.0 (C-1′), 69.0 (C-2),62.2 (C-1, C-3), 34.3 (C-2′), 34.2 (C2″, C-2‴), 32.1 (C-12′, C-12″, C-12‴),29.8-29.2 (overlapping signal C-4′, C-4″, C-4‴–C-11′, C-11″, C-11’”), 25.0(C-3′, C-3″, C-3’”), 22.8 (C-13′, C-13″, C-13‴), 14.2 (C-14, C-14″, C-14‴)(Supplementary Material S17–S18) (Jumina *et al*. 2018).

### Phytochemical Analysis

The stock solutions of methanol and ethyl acetate extracts of *H. polyspherula* were prepared to 1 mg/mL concentration in methanol. The stock solution was prepared by dissolving the 50 mg of the samples in 10 mL of methanol as stock solution used for the qualitative phytochemical screening of flavonoids, steroids, triterpenoids, tannins, saponins, alkaloids, phytosterol, resins, quinone and lignin, adopting the standard procedure with minimum modifications (Bitwell *et al*. 2023).

### Total Phenolics Content

The Folin-Ciocalteu reagent method was adopted and modified to determine the total phenolic content (Martins *et al*. 2021). Gallic acid (1.0 mg) was dissolved in 1.0 mL of methanol to create a stock serial solution with a concentration of 1.0 mg/mL for the standard. From the stock solution, subsequent dilutions of 0.2 mg/mL, 0.4 mg/mL, 0.6 mg/mL, 0.8 mg/mL and 1.0 mg/mL were produced. A 10% Folin solution was created by mixing 5.0 mL of the Folin reagent with 45 mL of distilled water. Furthermore, 5% sodium carbonate solution was prepared by mixing 1.5 g of sodium carbonate with 25 mL of distilled water. The sample (200 μL) was placed in a test tube, 1.5 mL of 10% Folin solution was added, and the test tube was left in dark for 5 min. The solution was thereafter thoroughly mixed with 1.5 mL of 5% sodium carbonate, the reaction was allowed at room temperature to run its course for 2 h in the dark. Using Shimadzu UV-2600 UV-Vis Spectrophotometer, the absorbance was measured at a wavelength of 750 nm. Gallic acid calibration curve was plotted, and the results were calculated using the calibration curve’s regression equation and expressed in milligrams of gallic acid equivalent per gramme of sample (mg GA/g), the total phenolic content was measured using Equation 1.


(1) $$\text{T}=\frac{C\times V}{M}$$

where *T* is the amount of sample extract in milligram per gramme of sample (mg GA/g). *C* is the amount of gallic acid in milligram, *V* is the extract’s volume (mL) and *M* is the weight in gram of the sample extract.

### Total Flavonoids Content

The colourimetric method was modified to generate the quercetin standard calibration curve for total flavonoids contents of ethyl acetate and methanol extracts of *H. polyspherula* (Shraim *et al*. 2021). Firstly, the calibration curve of the standard was done by dissolving 3.0 mg of quercetin in 3.0 mL of methanol to have a stock solution of 1.0 mg/mL. Serial concentrations of 0.2 mg/mL, 0.4 mg/mL, 0.6 mg/mL, 0.8 mg/mL and 1.0 mg/mL were prepared from the stock solution, similarly, the samples were dissolved in methanol and same serial dilutions were applied. Sodium nitrate (2.5 g) was dissolved in 50 mL of distilled water in volumetric flask to obtained of 5% NaNO_3_. Meanwhile, 4% solution of NaOH was prepared by mixing 3.0 g of NaOH and 75 mL of distilled water in a volumetric flask while 10% AlCl_3_ was prepared by dissolving 3.0 g in 30 mL of distilled water. The analysis was carried out by adding 250 μL of the quercetin in a test tube followed by 1,250 μL of distilled water and 750 μL of NaNO_3_, the mixture was then left in the dark for 6 min. Furthermore, 150 μL of AlC_13_, 500 μL (NaOH) and 275 μL of distilled water were added to the solution for completion of the reaction. The reaction was left in the dark for another 5 min. Using Shimadzu UV-2600 UV-Vis Spectrophotometer, the absorbance was measured at 510 nm wavelength. Results were ascertained using the regression equation of quercetin calibration curve. The total flavonoid content was expressed in milligrams of quercetin equivalent per gramme of sample (mg QE/g). The total flavonoid content was calculated using Quercetin equivalents (QE) using Equation 1.

### DPPH Radical Scavenging

The free radical scavenging activity of the samples was determined using 1,1-diphenyl-2 picrylhydrazyl (DPPH) assay as described with minor modification (Chua *et al*. 2013). The DPPH solution was prepared by dissolving 3.0 mg of DPPH in 150 mL of methanol to afford 20 μg/mL. A 750 μL of the sample’s solution at different concentrations, ranging from 1.25 mg/mL to 40 mg/mL was added to 1,500 μL of DPPH solution. The absorbance was recorded at 517 nm using Shimadzu UV-2600 UV-Vis spectrophotometer after 15 min of incubation at room temperature. Ascorbic acid was used as the positive control, while methanol was used as negative control for scanning the background. The ability to scavenge the DPPH was calculated using Equation 2:


(2) $$\text{DPPH radical scavenging activity }(\%)=\frac{\text{Abs control}-\text{Abs sample}}{\text{Abs control}}\times 100$$

where Abs control is the absorbance of DPPH while Abs sample is the absorbance of the sample. The concentration of sample required to scavenge 50% of DPPH radical (IC_50_) was determined based on the ascorbic acid calibration curve (0 mg/mL–10 mg/mL). All experiments were performed in triplicates.

### Acetylcholinesterase and Butyrylcholinesterase

To assess the inhibitory activity of *H. polyspherula* extract, fractions and isolated compounds towards AChE and BuChE, Ellman’s spectrophotometric method was followed (Ellman *et al*. 1961) using purified AChE from *Electrophorus electricus* (electric eel) Type V-S (C2888-1K), and BuChE from equine serum lyophilized powder (C7512-1.2KU) all from Sigma Aldrich, Germany. The reaction took place in a final volume of 200 μL of a (140 μL to 160 μL) 100 mM phosphate-buffered solution at pH 7.5 for AChE and 8.0 for BuChE, containing (20 μL) 0.2 U of AChE or 0.1 U of BuChE. Inhibition curves were made by pre-incubating this mixture with six concentrations of each sample (20 μL) for 15 min. A sample with no compound (blank) was present to determine the 100% of enzyme activity. After the pre-incubation period, ten microlitre (10 μL) of 10 mM of 5,5′-dithiobis-2-nitrobenzoic acid (DTNB, D8130 Sigma Aldrich, Germany) was added prior to 14 mM (10 μL) acetylthiocholine iodide (A5751) or 14 Mm butyrylthiocholine iodide (B3253-5G) source from Sigma Aldrich, Germany) were added, DTNB produces yellow anion 5-thio-2-nitrobenzoic acid along with the enzymatic degradation of ATChI or BuTChI. Changes in absorbance were detected at 412 nm in a 96 well spectrophotometric microplate reader (Bostch Biotek) after 30 min. Samples inhibiting AChE or BuChE activity would reduce the colour generation, thus, IC_50_ values were calculated as the concentration of compound that produces 50% AChE and BuChE activity inhibitions. Data are analysed using excel, graph pad prism 9.0.0 and expressed as means ± SD of at least three different experiments in triplicate.


(3) $$\%\;\text{inhibition}=\frac{\text{Abs }(\text{blank})\times \text{Abs }(\text{inhibitor})}{\text{Abs }(\text{blank})}\times 100$$

### Statistical Analysis

The results were analysed in triplicates (*n* = 3) using Microsoft Excel 2016, IBM SPSS Statistics 23 and Graph pad prism 9.0.0 and the means are expressed corresponding to their standard deviation for all the samples.

## RESULTS AND DISCUSSSION

The EtOAc and methanol extracts of bark of *H. polyspherula* were submitted to BuChE/AChE assay using Ellman’s spectrophotometric method (Table 1). EtOAc extract demonstrated superior inhibition of 77% and 64% against AChE and BuChE, respectively, while the methanol extract showed an inhibition of 38% and 39%, respectively, these extracts subjected to CC and the fractions were further analysed for their anti-cholinesterase and anti-butyrylcholinesterase activities (Kaneria & Chanda 2013). Fractions Fr3 and Fr7–Fr10 exhibited strong inhibitions above 50%, while fraction Fr4 showed weak potency with inhibitions of less than 50% against both enzymes. Fractions Fr5–Fr6 and Fr11 showed monoenzymatic inhibition against either AChE or BuChE, with inhibition of more than 50%, contrary to fractions Fr1 and Fr2, which did not show any activity against both enzymes at the tested concentrations.

**Figure 1:**
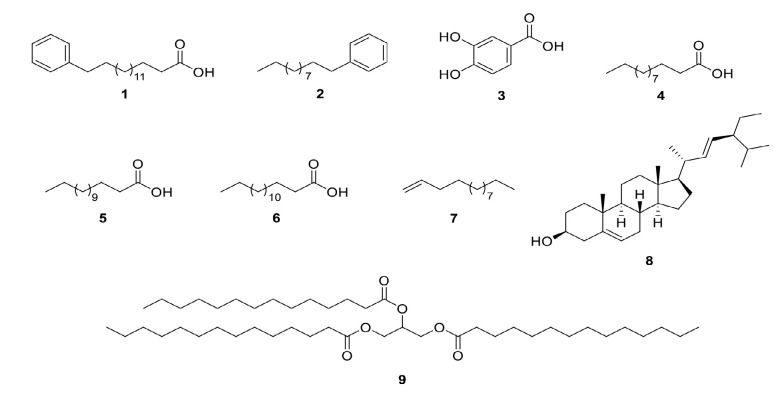
Chemical structures of isolated compounds **1–9** from *H. polyspherula*.

EtOAc extract had clearly showed the best inhibitory activities on butyrylcholinesterase and acetylcholinesterase, this extract was subjected to silica gel column chromatography, affording eleven fractions designated as Fr.1–Fr.11. Nine compounds were isolated namely 16-phenylhexadecanoic acid (**1**), undecylbenzene (**2**), 3,4-dihydroxybenzoic acid (**3**), dodecanoic acid (**4**), tetradecanoic acid (**5**), pentadecanoic acid (**6**), 1-tridecene (**7**), stigmasterol (**8**) and trimyristin (**9**). The structures of these compounds were thoroughly investigated via assignment with 1D-NMR, 2D-NMR, FT-IR and HRESIMS as indicated in Fig. 1.

The qualitative phytochemical screening indicated the presence of flavonoids, steroids, lignins and alkaloids (Iodine test) in extracts, while tannins, saponins, quinones, resins, alkaloids (Dragendroff’s reagent) and phytosterol (acetic anhydride test) were absent, meanwhile phytosterol (Hess’s test), alkaloid (Wagner’s test) and triterpenoids were observed in both MeOH and EtOAc extracts respectively as presented in Table 2.

The quantitative analysis for the total flavonoids content in the extracts were shown in Table 3, the results revealed the amount expressed in milligram per gram quercetin equivalent. More so, the quantitative analysis for the phenolic content was expressed as gallic acid equivalent, the DPPH IC_50_ value (define as the concentration of test material required to scavenge 50% free radical) of the extracts and Compounds **3** and **9** isolated from *H. polyspherula* bark were determined by the plotted graph of concentration against the corresponding scavenging activity (Supplementary Material S19). The lowest IC_50_ indicates the strongest ability of the test material to act as DPPH radical scavenger.

A concentration dependent study with ascorbic acid as positive control revealed that the IC_50_ value of ethyl acetate extract, methanol and Compound **3** 176 were 8.94 ± 0.41 μg/mL, 7.27 ± 0.25 μg/mL and 6.56 ± 0.02 μg/mL, respectively (Table 4). The higher IC_50_ exhibited by both extracts and Compound **3** indicate their effectiveness as DPPH radical scavengers, and thus were potent antioxidant agents (Mohamed Yusoff *et al*. 2022). Statistically, the IC_50_ showed a significance difference at *p* < 0.05 about the group using Tukey HSD one-way ANOVA. IC_50_ being the reflection of % inhibition, Compound **9** show no activity at the analysed concentration, hopefully at higher concentration above 40 μg/mL might be active for DPPH radical. Furthermore, this appreciable scavenging activity exhibited by methanol extract could be attributed to the maximum total flavonoids content (137.25 ± 24.06 mg GEA/g) as well as the total phenolic content (619 ± 1.74 mg QE/g).

The scavenging activity of EtOAc extract was connected to its phenolic content (364.83 ± 1.51 GAE mg/g) and non-phenolics such as lower fatty acids. Additionally, the EtOAc extract also exhibited (Refer Supplementary Material S20 for details) a high amount of quercetin equivalent (116.67 ± 16.98 mg QE/g) which agrees with the reported flavonoids isolated from EtOAc fraction of *H. superba* (Al-Mekhlafi *et al*. 2013). This may in turn reflects that flavonoids present in *Horsfieldia* species are richly found in EtOAc extract, as such validate the good percentage antioxidant observed in current research. Statistically, one-way anlysis of varience using Tukey HSD (*p* < 0.05) indicated no signifance difference between the flavonoids content of the two extracts while significance difference was observed within the total phenolic content and DPPH between the samples (*p* < 0.05).

The IC_50_ results for the inhibition potential of the EtOAc extract, MeOH extract and the nine compounds against BuChE/AChE were evaluated for only four compounds due to limited quantity of the isolated compounds. Table 5 displays the IC_50_ values of the assayed compounds analysed at different concentrations (40 μM–1000 μM). They possessed higher IC_50_ values compared to the positive control (eserine) (Supplementary Material S21–S22) at concentrations ranging from 0.2 μM–15 μM). This could be associated with lack of carbamate or indole moieties as compared to eserine. The outstanding activity displayed by EtOAc (BuChE IC_50_ = 15.41 ± 0.78 μg/mL and AChE IC_50_ = 7.67 ± 0.13 μg/mL) (Supplementary Material S23–S24) could be attributed to either yet to be isolated compounds or cumulative activities of all compounds in the extract as well as the antioxidant activity shown by the extract against DPPH radical scavenger (Sedem *et al*. 2024). Furthermore, Compound **1** exhibited exceptional inhibitory activity against AChE (46.83 ± 2.44 μM) and BuChE (40.99 ± 1.99 μM), followed by Compound **2** (AChE 246.12 ± 1.40 μM; BuChE 233.79 ± 4.78 μM) and Compound **3** (AChE 327.36 ± 6.83 μM; BuChE 231.97 ± 8.37 μM), while Compound **9** has no inhibitory activities against both enzymes, as evidenced by the IC_50_ value, presented in Supplementary Materials S25 to S26.

Structurally, Compound **1** has a phenyl group, alkyl group and carbonyl group, unlike Compound **2** that has phenyl and alkyl groups (Gonfa *et al*. 2023; Sardar *et al*. 2022). Furthermore, Compound **3** has phenyl, carbonyl and two hydroxyl groups, while Compound **9** has a carbonyl group, but in the form of an ester (Ourhzif *et al*. 2022). The activity of Compound **1** could be associated with the cumulative activity of the 15 alkyl groups, phenyl and acid carbonyl groups in the compound, which can make a hydrogen and alkyl-alkyl bond with the enzyme Compound **2** has the advantage of 11 alkyl groups, which are vital in the formation of alkyl-alkyl bonds between the compound and the enzyme as well as possible activity of the terminal alkyl group with the enzyme. Compound **3** lacks an alkyl group but has a carbonyl group that could hinders the activity of the two hydroxyl groups attached to the phenyl group, which in turn may results in minimal activity against these enzymes. On the other hand, the structure of Compound **9** could contribute to its unfavourable effect against cholinesterase and thus render it inactive (Omidpanah *et al*. 2020). The selectivity index acts as indicator for predicting how enzymes to particular biological inhibitors (Liu *et al*. 2024). The selectivity index of both extracts and Compounds **1**–**3** against AChE and BuChE afforded selectivity values ranging between 0.7 to 2.1 for AChE and 0.5 and 1.5 for BuChE (Table 5). 178

## CONCLUSION

This study is centered on the qualitative phytochemical analysis of EtOAc and MeOH extracts, the quantification of total phenolic and flavonoid content, assessment of antioxidant activity and the evaluation of *in vitro* cholinesterase inhibition properties of *Horsfieldia polyspherula*. Employing a bioassay-guided approach with Ellman’s spectroscopic method, nine compounds were isolated from the EtOAc extract. Notably, 16-phenylhexadecanoic acid (**1**), undecylbenzene (**2**), pentadecanoic acid (**6**) and 1-tridecene (**7**) were identified for the first time within the *Horsfieldia* genus and the broader Myristicaceae family. Stigmasterol (**8**) was reported for the first time in the *Horsfieldia* genus, while 3,4-dihydroxybenzoic acid (**3**), dodecanoic acid (**4**), tetradecanoic acid (**5**) and trimyristin (**9**) had been previously documented in other *Horsfieldia* species. Compounds **1**–**3** were subjected to *in vitro* testing, where Compound **1** exhibited highest dual inhibitory activity against AChE/BuChE, albeit to a lesser extent compared to the positive control.

## Supplementary Information



## Figures and Tables

**Table 1:** Percentage of inhibition of *H. polyspherula* against BuChE and AChE at 100 μg/mL.

Extract/Fraction	% inhibition	

	BuChE	AChE
EtOAc extract	64.13 ± 1.02	77.18 ± 0.67
Fr. 1	>100	>100
Fr. 2	>100	>100
Fr. 3	77.99 ± 0.23	94.47 ± 0.26
Fr. 4	13.64 ± 0.65	31.47 ± 1.29
Fr. 5	52.80 ± 0.45	43.81 ± 1.07
Fr. 6	43.30 ± 0.79	74.93 ± 0.35
Fr. 7	87.13 ± 0.23	86.23 ± 1.14
Fr. 8	86.48 ± 0.31	71.49 ± 1.19
Fr. 9	89.62 ± 0.40	55.43 ± 0.50
Fr. 10	58.27 ± 0.12	65.66 ± 1.75
Fr. 11	57.94 ± 1.21	39.59 ± 0.00
MeOH extract	39.24 ± 1.01	37.50 ± 0.48

Eserine (Control, tested at 0.2 μM)	44.45 ± 0.23	76.61 ± 1.31

**Table 2:** Results for the phytochemical screening of the plants.

Phytochemical	*H. polyspherula*	

	MeOH extract	EtOAc extract
Flavonoid	+	+
Steroid	+	+
Triterpenoid	−	+
Tannins	−	−
Saponins	−	−
Alkaloid		
i. Wagner’s test	+	−
ii. Dragendroff’s test	−	−
iii. Iodine test	+	+
Phytosterol		
i. Acetic anhydride	−	−
ii. Hess’s response	+	−
Resins		
i. Acetic anhydride test	−	−
ii. Turbidity test	−	−
Quinone	−	−
Lignins	+	+

*Note*: Absent (−), Present (+)

**Table 3:** GEA and QE for ethyl acetate and methanol extracts of *H. polyspherula*.

Sample	Total flavonoids content (mg QE/g extract)	Total phenolic content (mg GAE/g extract)
EtOAc extract	116.67 ± 16.98	364.83 ± 1.51
MeOH extract	137.25 ± 24.06	619.00 ± 1.74

*Note*: Values mean significant difference( *p* < 0.05); mean ± SD, *n* = 3

**Table 4:** IC_50_ value of DPPH free radical scavenging activity for the extracts and compounds isolated from *H. polyspherula* bark.

Sample	IC_50_ (μg/mL)
EtOAc extract	8.94 ± 0.41
**3**	6.56 ± 0.02
**9**	> 400
MeOH extract	7.27 ± 0.25
Ascorbic acid	4.21 ± 0.13

*Note*: Values mean significant difference( *p* < 0.05); mean ± SD, *n* = 3

**Table 5:** IC_50_ of ethyl acetate extract, methanol extract and compounds isolated from *H. polyspherula* ethyl acetate extracts against BuChE and AchE.

Sample	IC_50_ (μg/mL or μM)			

	BuChE	SI	AChE	SI
EtOAc extract	15.41 ± 0.78	0.50	7.67 ± 0.13	2.01
1	40.99 ± 1.99	1.14	46.83 ± 2.44	0.88
2	233.79 ± 4.78	1.05	246.12 ± 1.40	0.95
3	231.97 ± 8.37	1.41	327.36 ± 6.83	0.71
MeOH extract	61.69 ± 0.24	0.77	47.60 ± 1.27	1.30

Eserine (Control)	0.47 ± 0.00	0.15	0.07 ± 0.00	6.71

*Notes*: μg/mL for extract and μM for the pure compound; selectivity index for AChE is define as IC_50_ (BuChE)/ IC_50_ (AChE); selectivity index for BuChE is defined as IC_50_ (AChE)/ IC_50_ (BuChE)
