# Supplementary material for: Phytochemical Analysis, Antioxidant Activity and Bioassay-Guided Isolation of Acetylcholinesterase and Butyrylcholinesterase Inhibitors from Horsfieldia polyspherula Bark (Myristicaceae)
Source: Trop Life Sci Res. 2024 Oct 7;35(3):165–83. doi: 10.21315/tlsr2024.35.3.8 (PMC11507974; doi:10.21315/tlsr2024.35.3.8)
Supplement: Supplementary file 1 [file TLSR_35-3-165-Suppl.pdf]

SUPPLEMENTARY MATERIAL

Phytochemical analysis, antioxidant activity and bioassay-guided isolation of acetylcholinesterase and butyrylcholinesterase inhibitors from *Horsfieldia polyspherula* stem bark (Myristicaceae)

[1,2]Mohammed Idris, [1]Mohamad Nurul Azmi*, [3]Thaigarajan Parmusivam, [4]Unang Supratman, [5]Marc Litaudon and [6]Khalijah Awang

[1]Natural Products and Synthesis Organic Research Laboratory (NPSO), School of Chemical Sciences, Universiti Sains Malaysia, 11800 Minden, Penang, Malaysia
[2]Department of Chemistry, Federal University, Gashua, Nigeria.
[3]School of Pharmacy, Universiti Sains Malaysia, 11800 Minden, Penang, Malaysia
[4]Department of Chemistry, Faculty of Mathematics and Natural Sciences, Universitas Padjadjaran, 45363 Jatinangor, Indonesia
[5]Institut de Chimie des Substances Naturelles, CNRS-ICSN UPR 01, Univ. Paris-Sud 11, Av. de la Terrasse, 91198 Gif-sur-Yvette, France
[6]Department of Chemistry, Faculty of Science, University of Malaya, 50603 Kuala Lumpur, Malaysia

*Corresponding author: mnazmi@usm.my

S1: ^{1}H NMR spectrum for compound **1** in $CDCl_3$ at 500 MHz	3
S2: ^{13}C NMR spectrum for compound **1**	4
S3: ^{1}H NMR spectrum for compound **2** in $CDCl_3$ at 500 MHz	5
S4: ^{13}C NMR spectrum for compound **2**	6
S5: ^{1}H NMR spectrum for compound **3** in CD_3OD at 500 MHz	7
S6: ^{13}C NMR spectrum for compound **3**	8
S7: ^{1}H NMR spectrum for compound **4** in $CDCl_3$ at 500 MHz	9
S8: ^{13}C NMR spectrum for compound 4	10
S9: ^{1}H NMR spectrum for compound **5** in $CDCl_3$ at 500 MHz	11
S10: ^{13}C NMR spectrum for compound **5**	12
S11: ^{1}H NMR spectrum for compound **6** in $CDCl_3$ at 500 MHz	13
S12: ^{13}C NMR spectrum for compound **6**	14
S13: ^{1}H NMR spectrum for compound **7** in $CDCl_3$ at 500 MHz	15
S14: ^{13}C NMR spectrum for compound **7**	16
S15: ^{1}H NMR spectrum for compound **8** in $CDCl_3$ at 500 MHz	17
S16: ^{13}C NMR spectrum for compound 8	18
S17: ^{1}H NMR spectrum for compound **9** in $CDCl_3$ at 500 MHz	19
S18: ^{13}C NMR spectrum for compound **9**	20
S19: Graphical resemblance of percentage scavenging activity between tested samples	21
S20: Graphical presentation of total phenolic and total flavonoid contents of the extracts of *H.* polyspherula bark. Values are expressed as mean ± standard deviation (n = 3).	22
S21: Graphical presentation of IC_{50} *eserine* (positive control) against Butyrylcholinesterase	23
S22: Graphical presentation of IC_{50} *eserine* (positive control) against Acetylcholinesterase	24
S23: Graphical relationship of IC_{50} of *H. polyspherula* extracts against Butyrylcholinesterase	25
S24: Graphical relationship of IC_{50} of *H. polyspherula* extracts against Acetylcholinesterase	26
S25: Graphical relationship of IC_{50} of *H. polyspherula* compounds against Butyrylcholinesterase	27
S26: Graphical relationship of IC_{50} of *H. polyspherula* compounds against Acetylcholinesterase	28

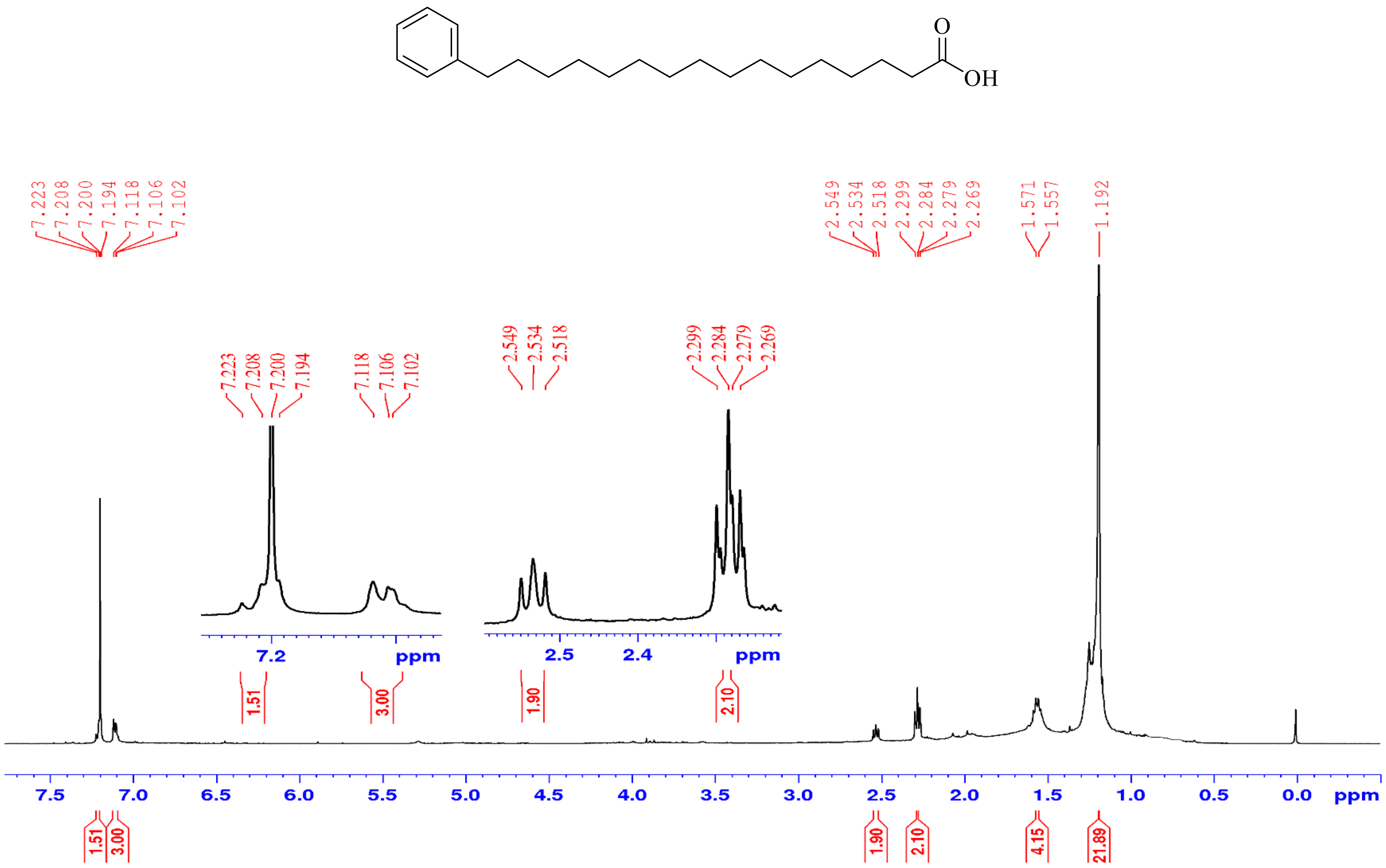

S1: ^{1}H NMR spectrum for compound **1** in $CDCl_3$ at 500 MHz

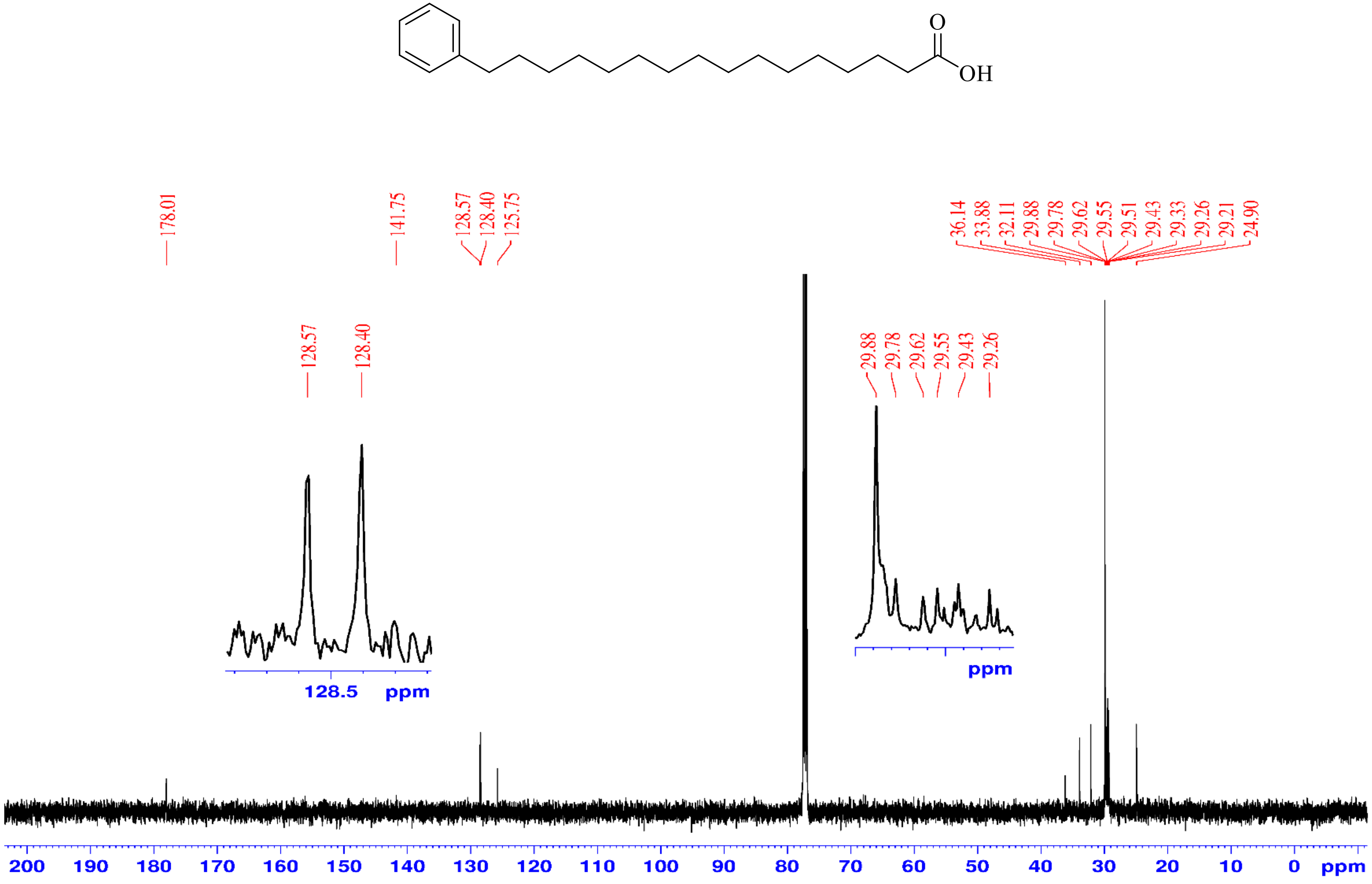

S2: ^{13}C NMR spectrum for compound **1**

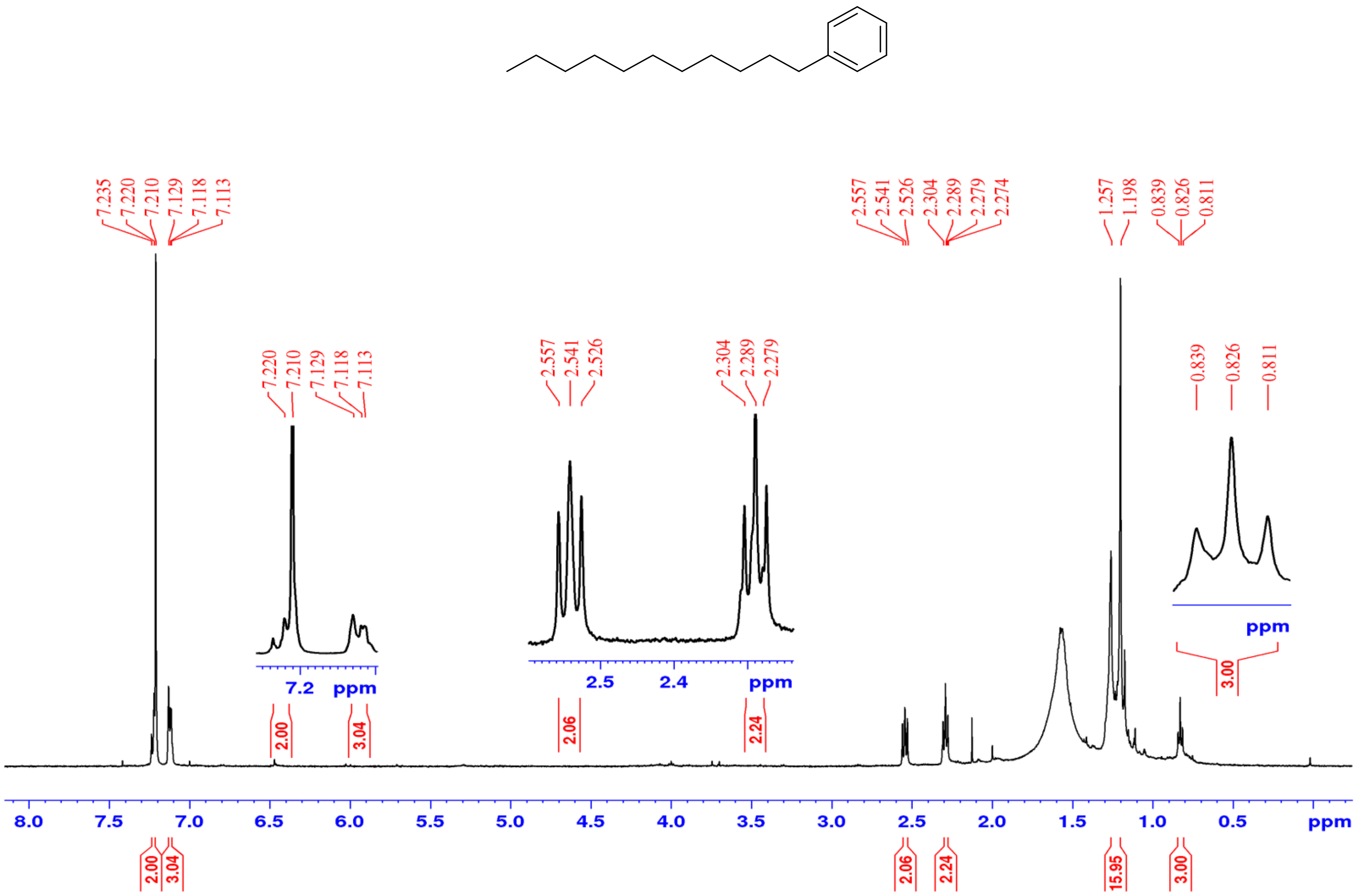

S3: ^{1}H NMR spectrum for compound **2** in $CDCl_3$ at 500 MHz

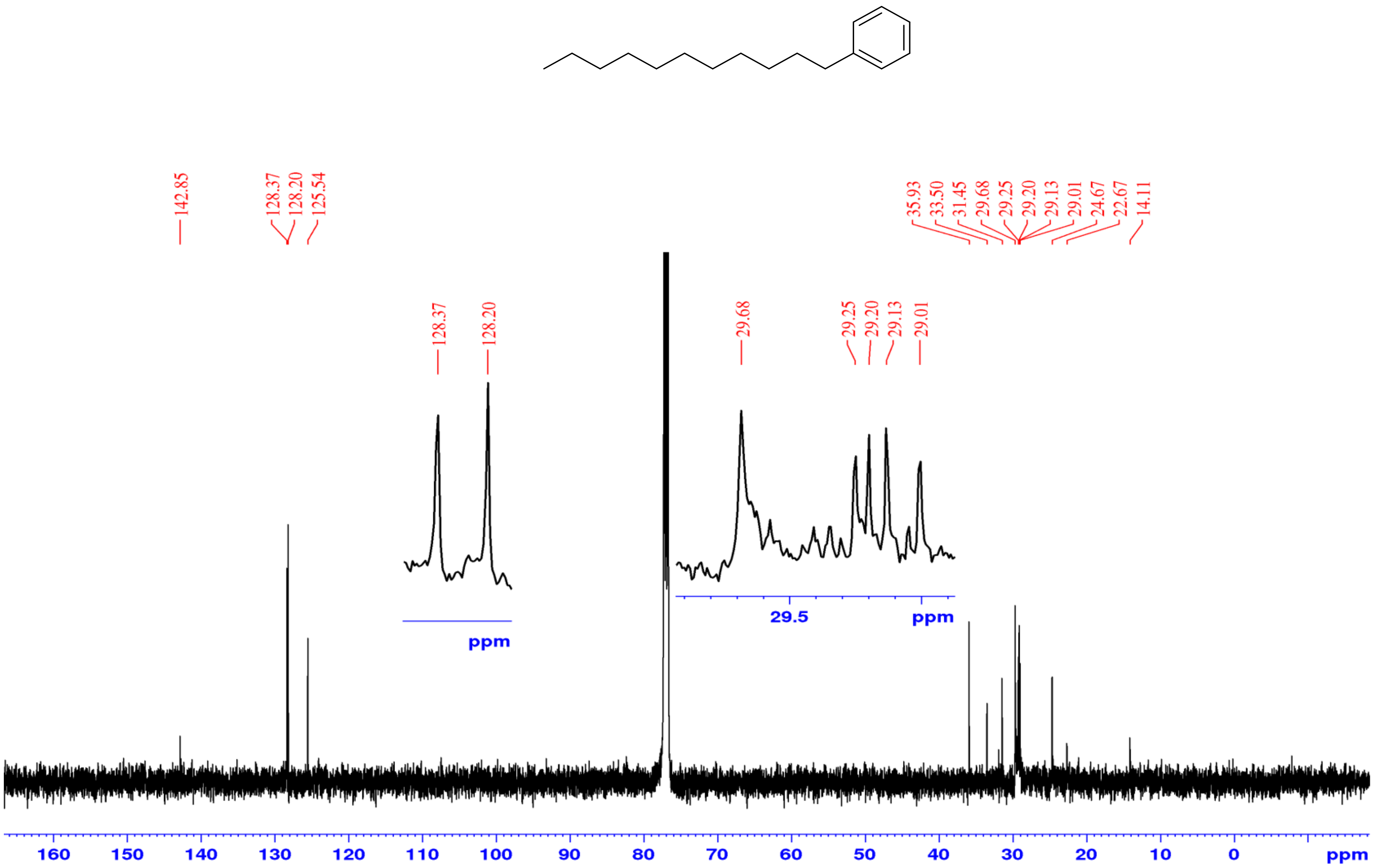

S4: ^{13}C NMR spectrum for compound **2**

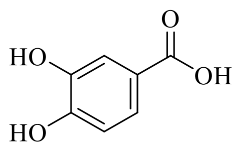

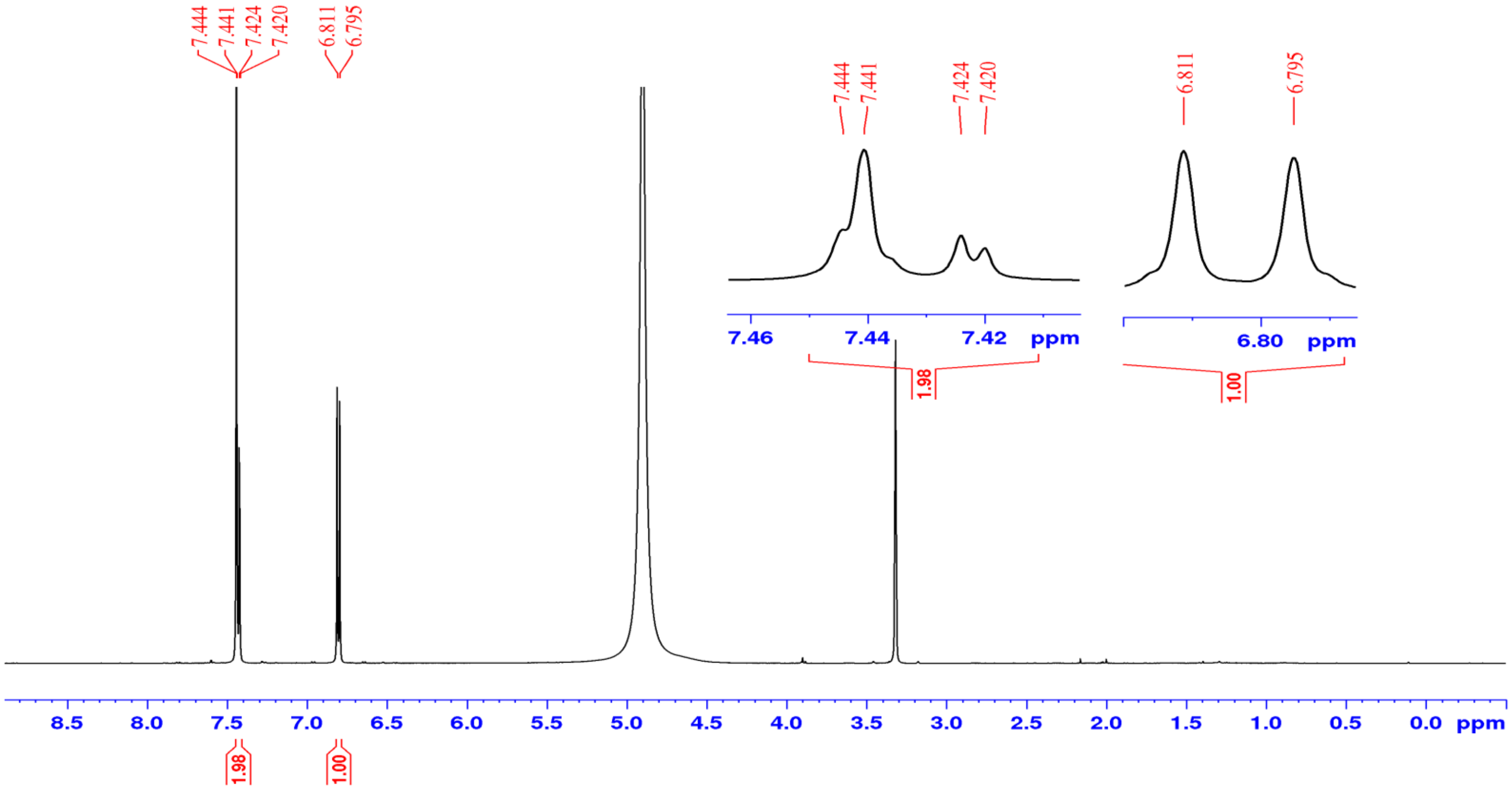

S5: ^1H NMR spectrum for compound **3** in CD_3OD at 500 MHz

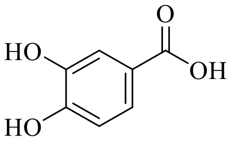

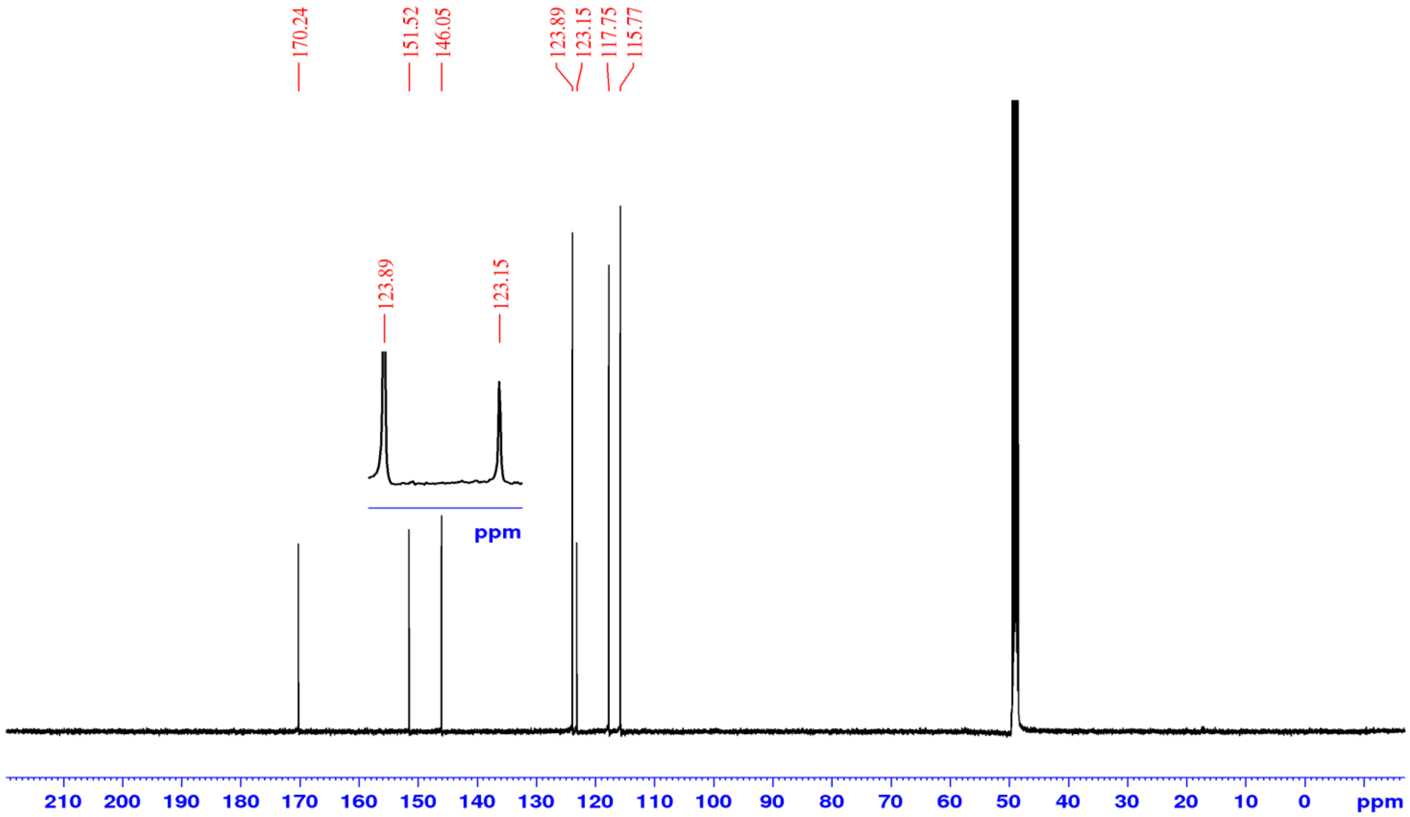

S6: ^{13}C NMR spectrum for compound **3**

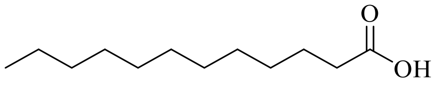

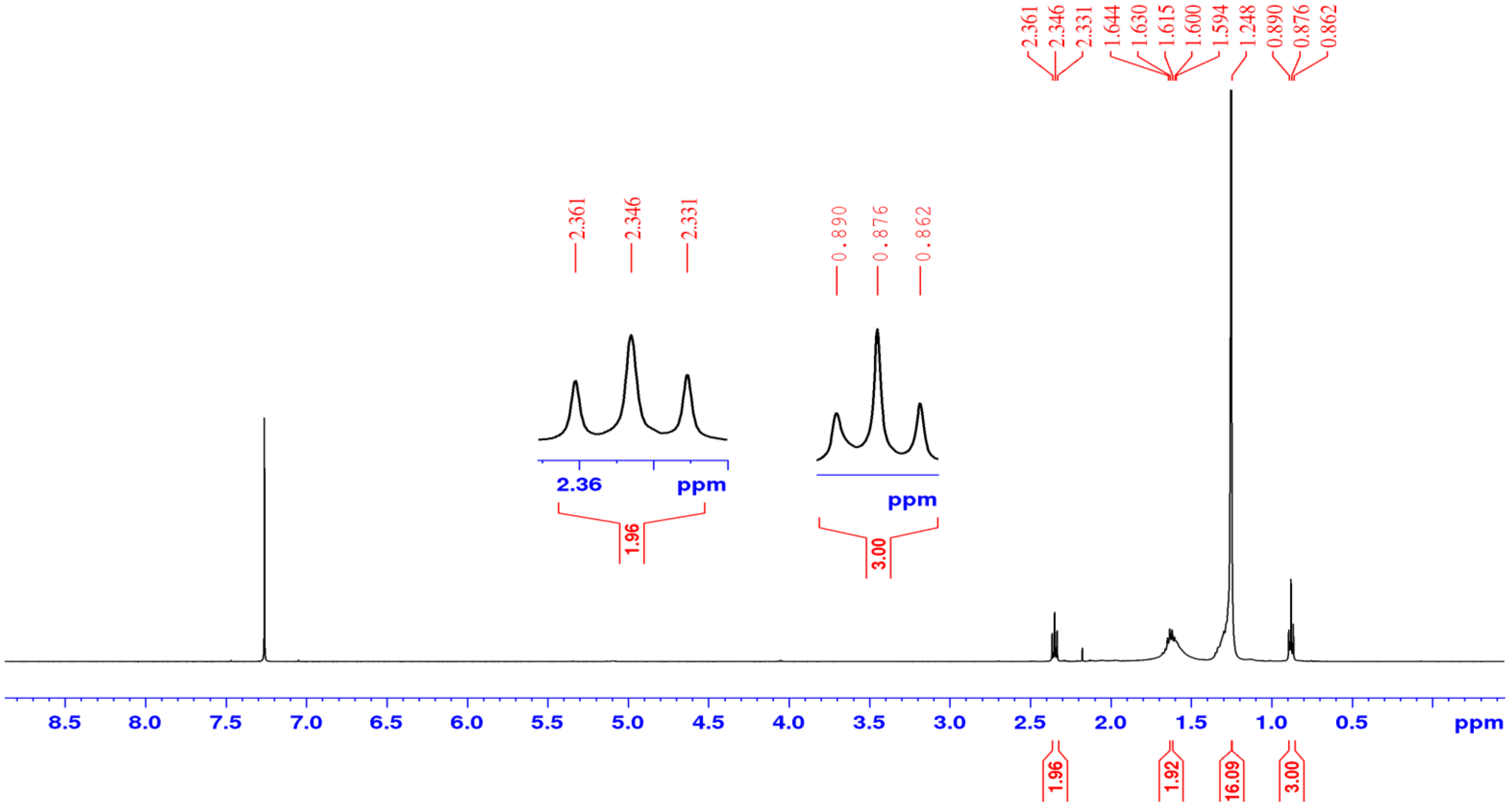

S7: ^{1}H NMR spectrum for compound **4** in $CDCl_3$ at 500 MHz

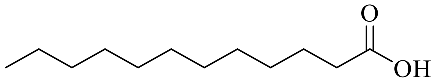

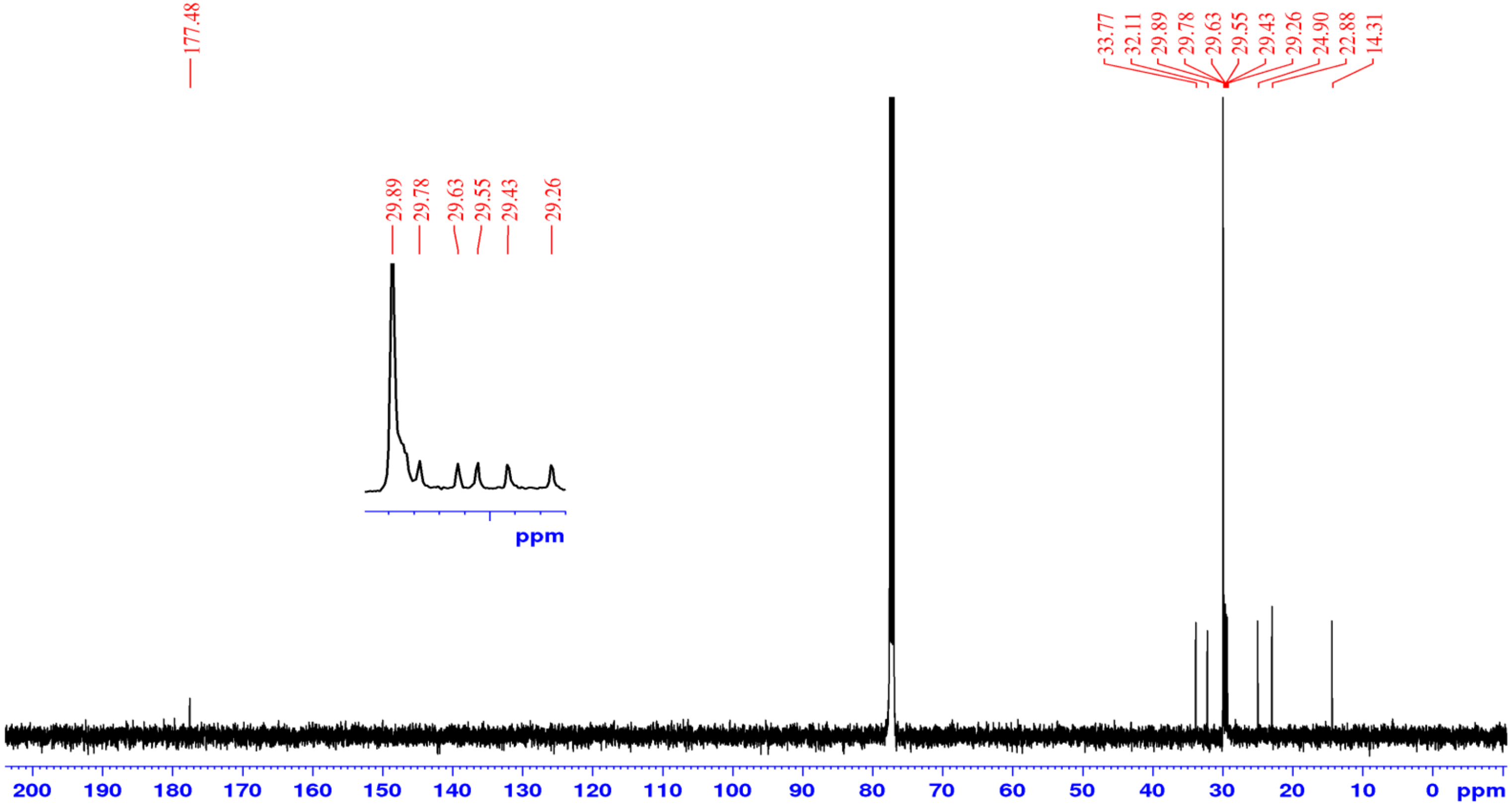

S8: ^{13}C NMR spectrum for compound **4**

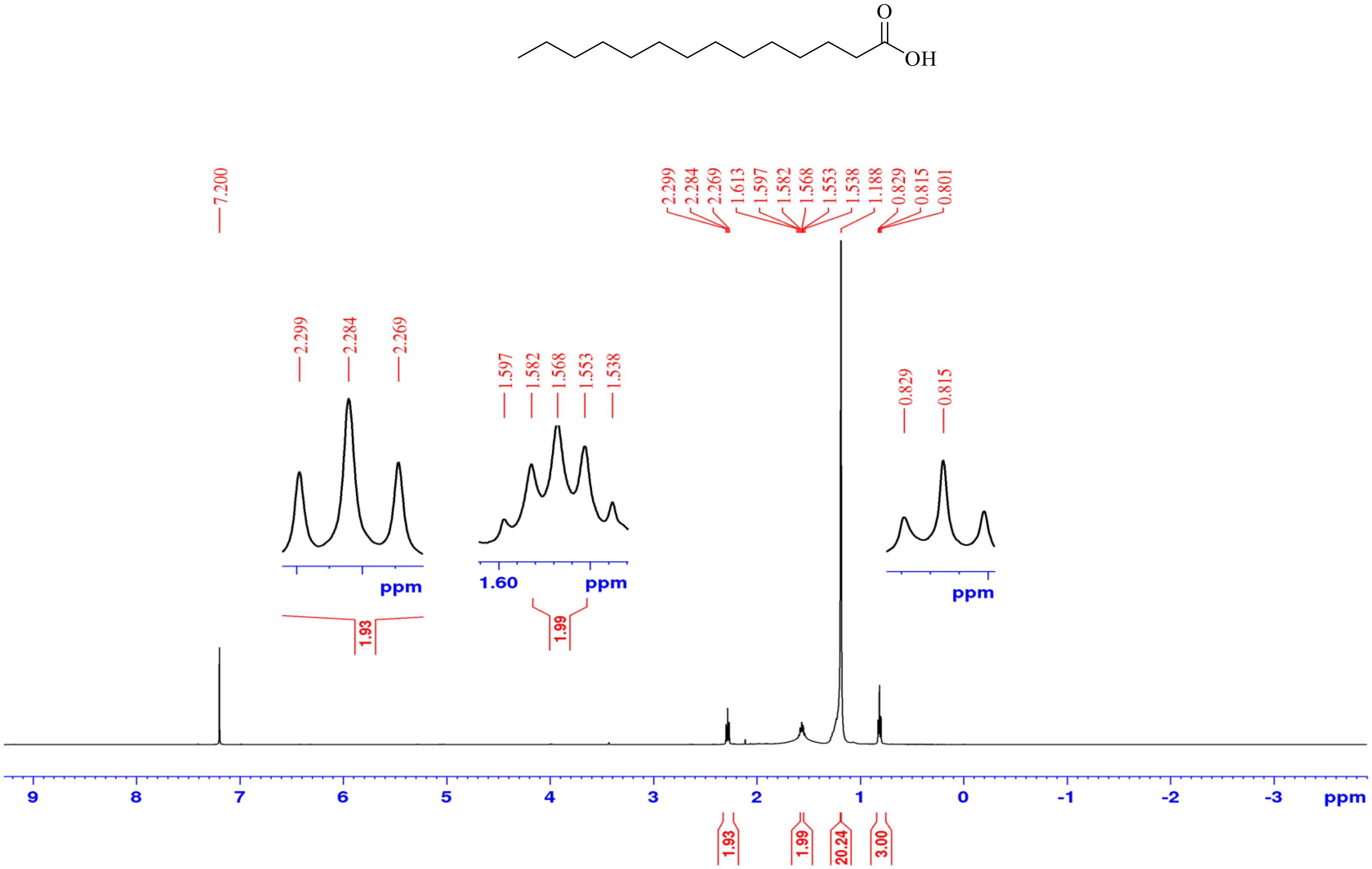

S9: ^{1}H NMR spectrum for compound **5** in $CDCl_3$ at 500 MHz

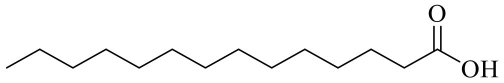

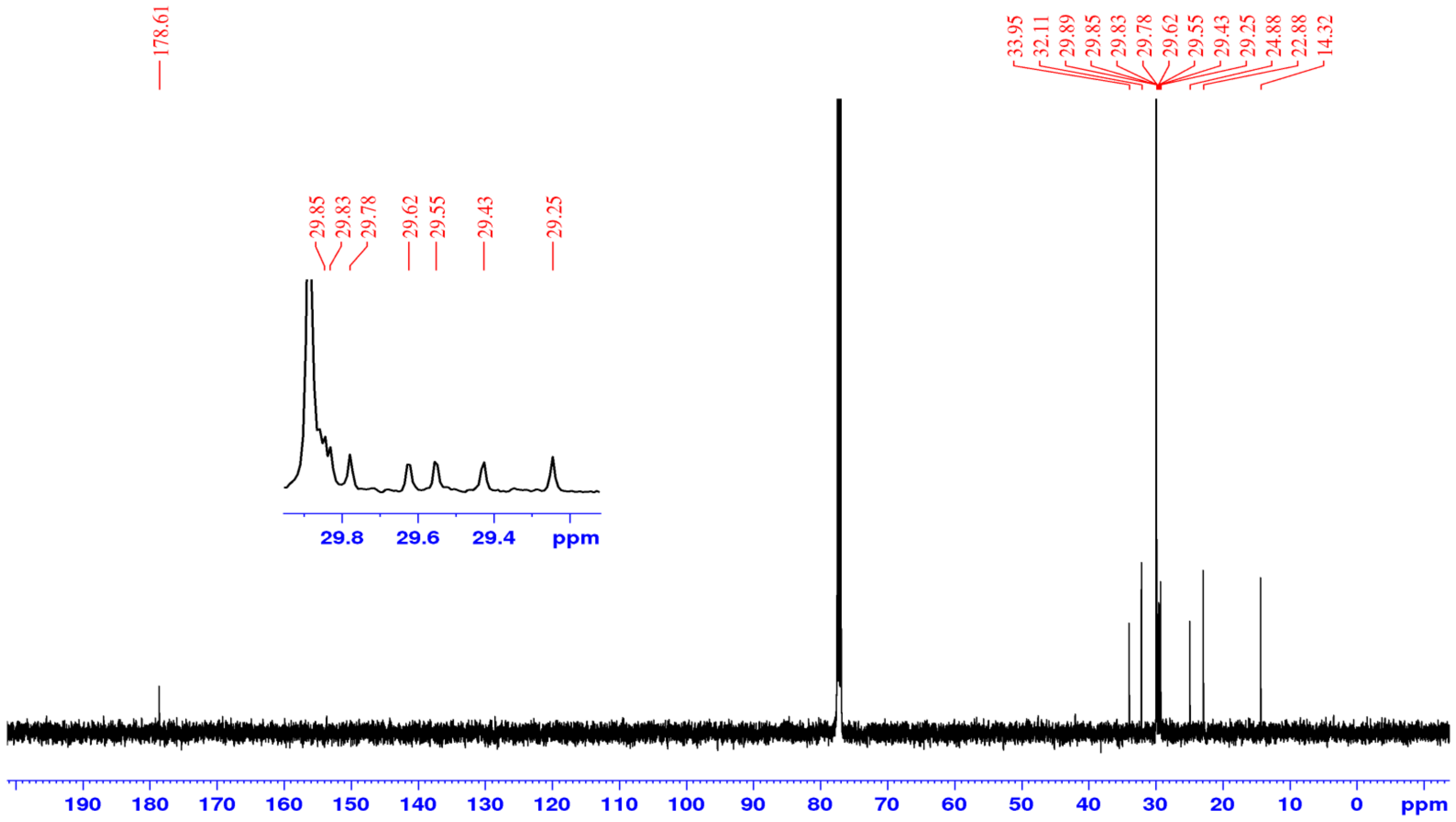

S10: ^{13}C NMR spectrum for compound **5**

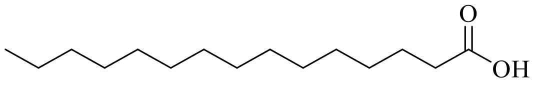

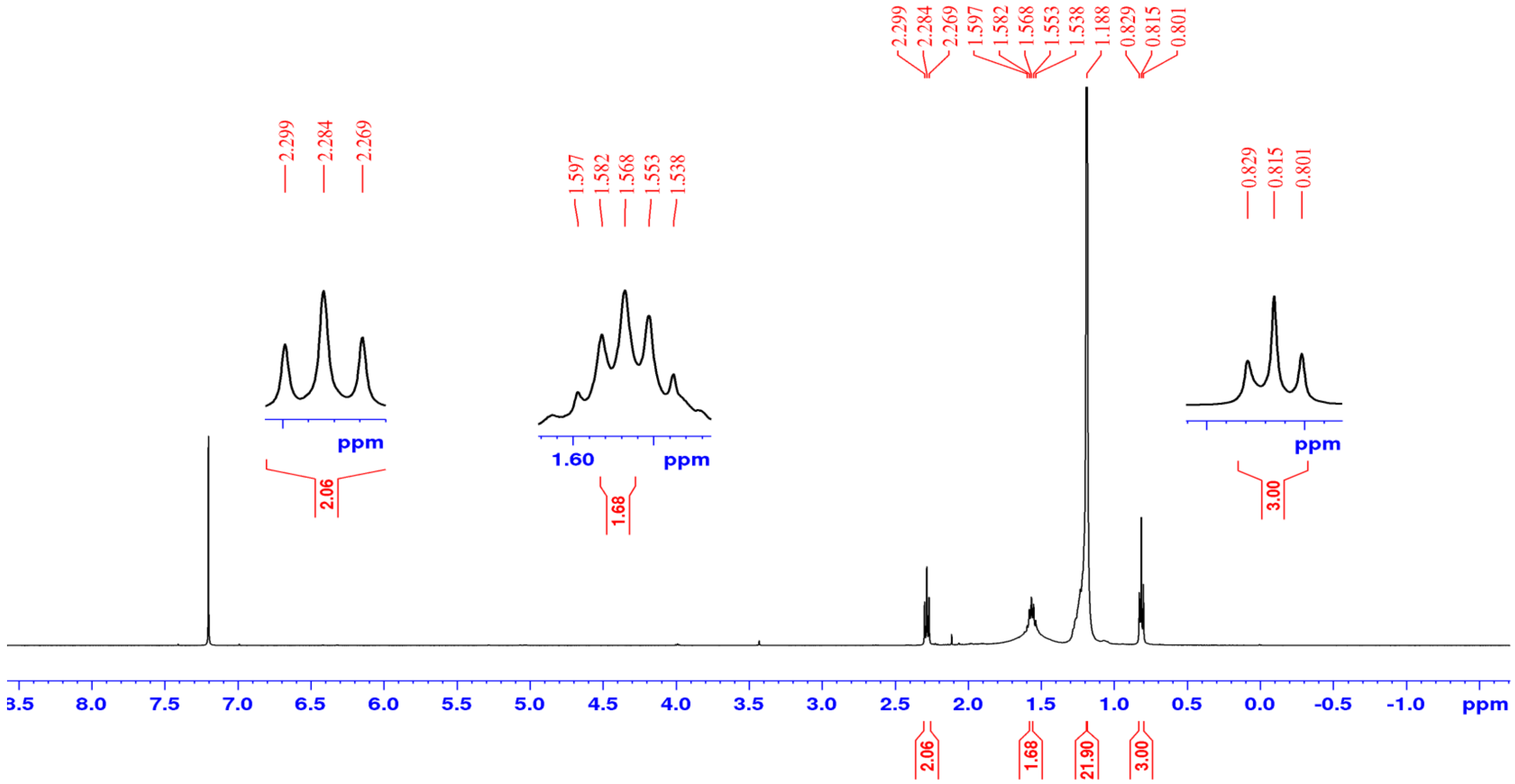

S11: ^{1}H NMR spectrum for Compound **6** in $CDCl_3$ at 500 MHz

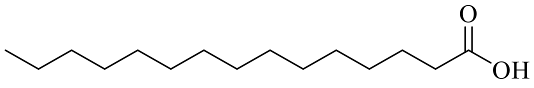

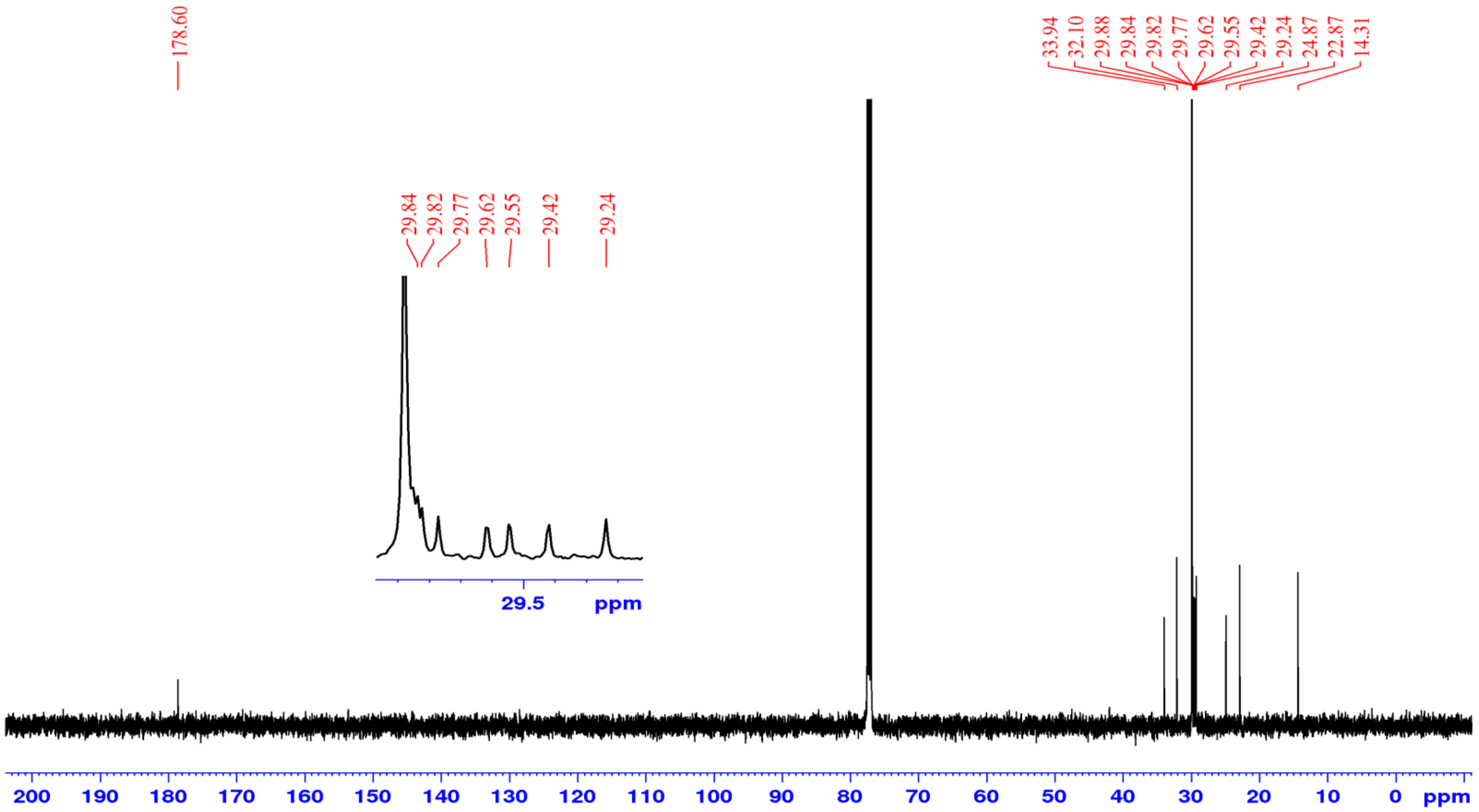

S12: ^{13}C NMR spectrum for **6**

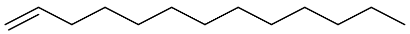

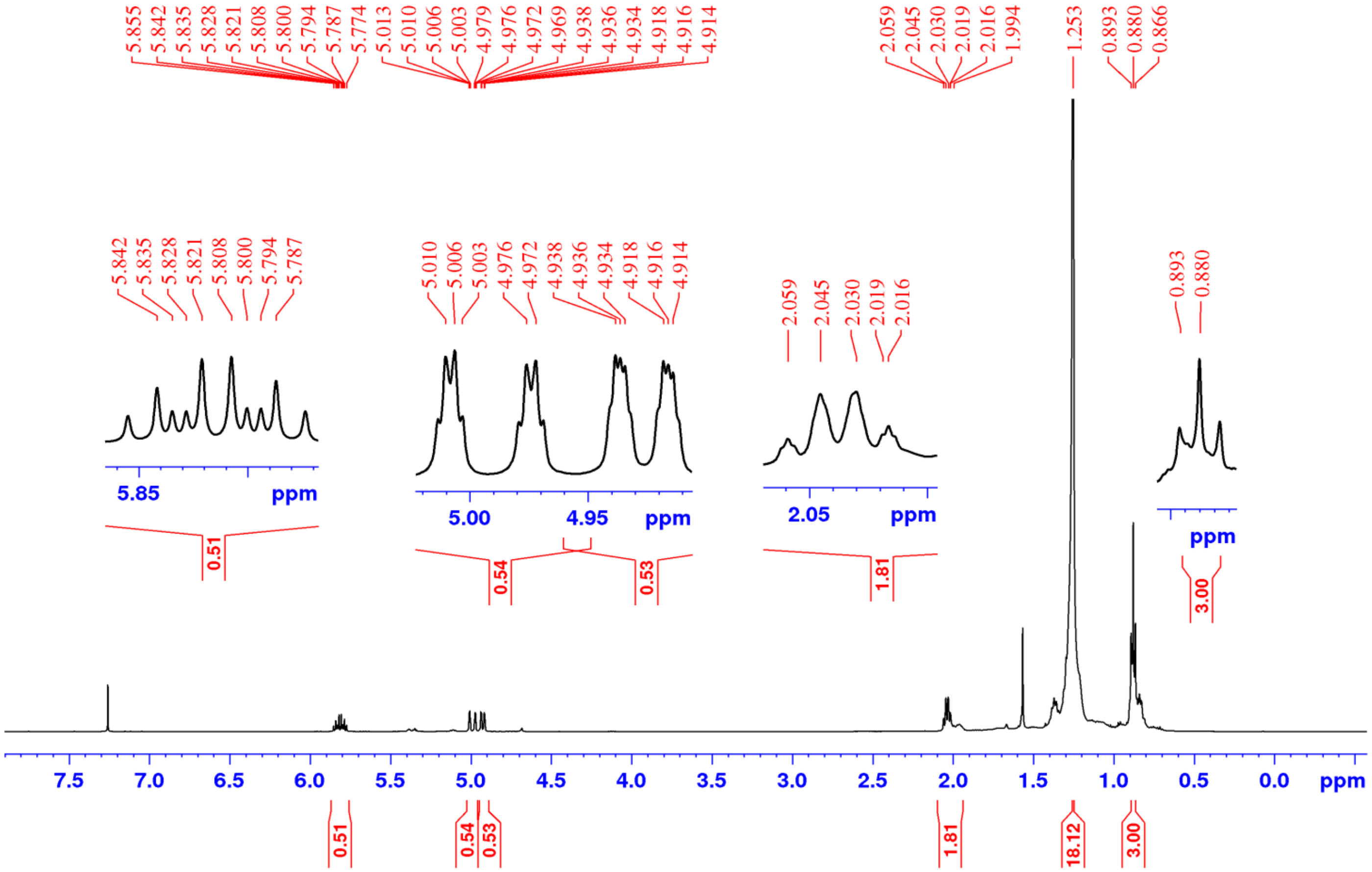

S13: ^{1}H NMR spectrum for compound **7** in $CDCl_3$ at 500 MHz

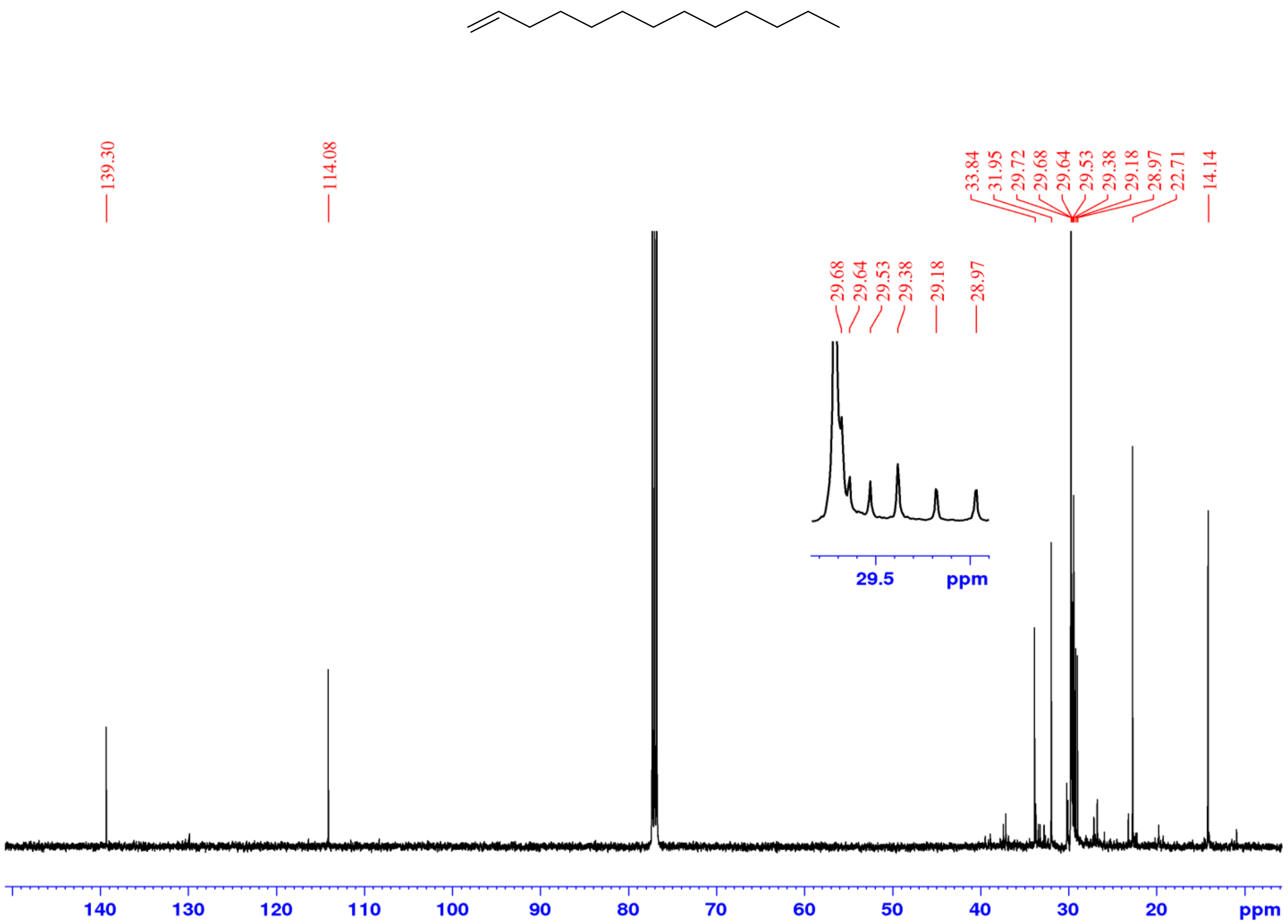

S14: ^{13}C NMR spectrum for compound **7**

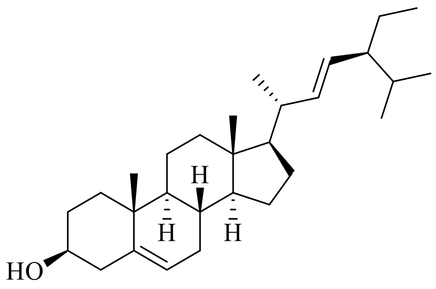

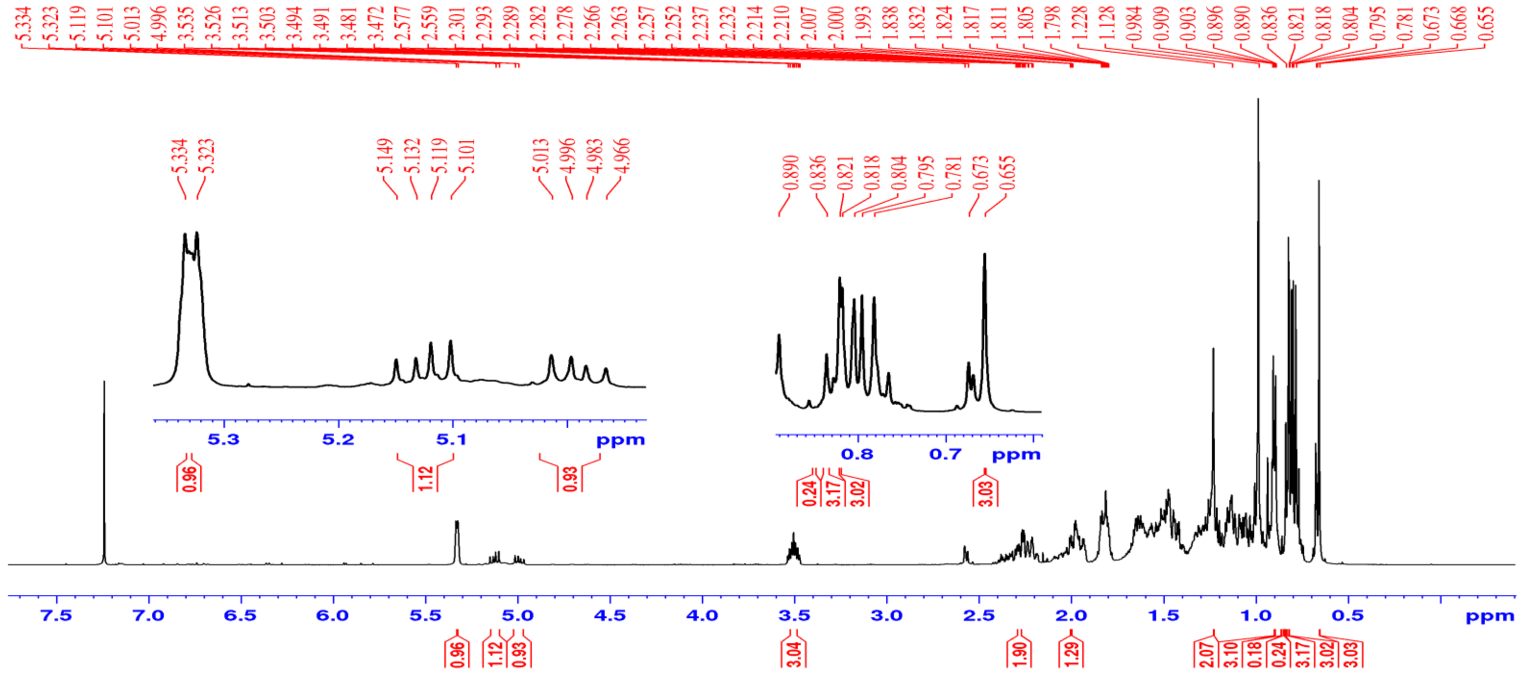

S15: ^{1}H NMR spectrum for compound **8** in $CDCl_3$ at 500 MHz

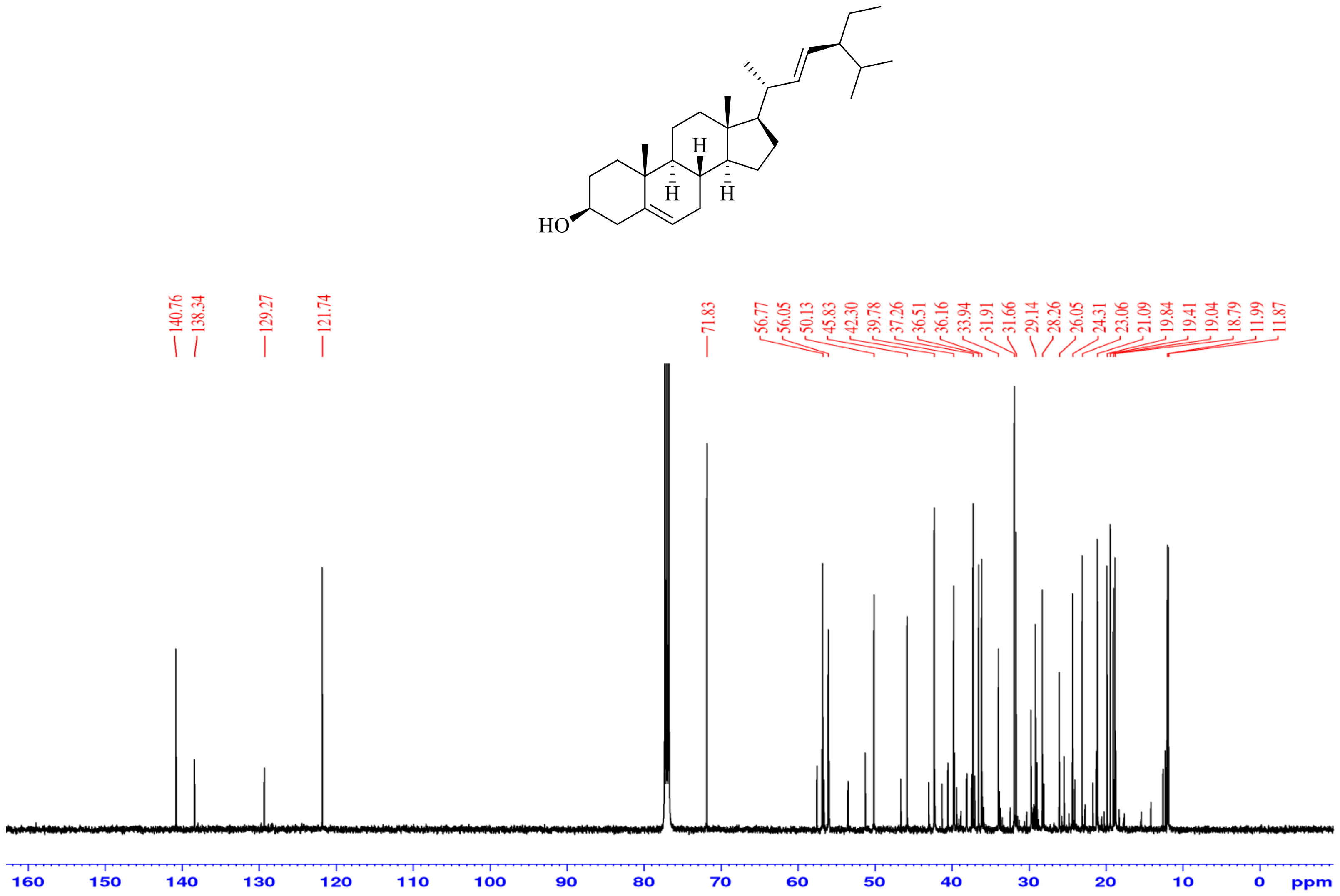

S16: ^{13}C NMR spectrum for compound **8**

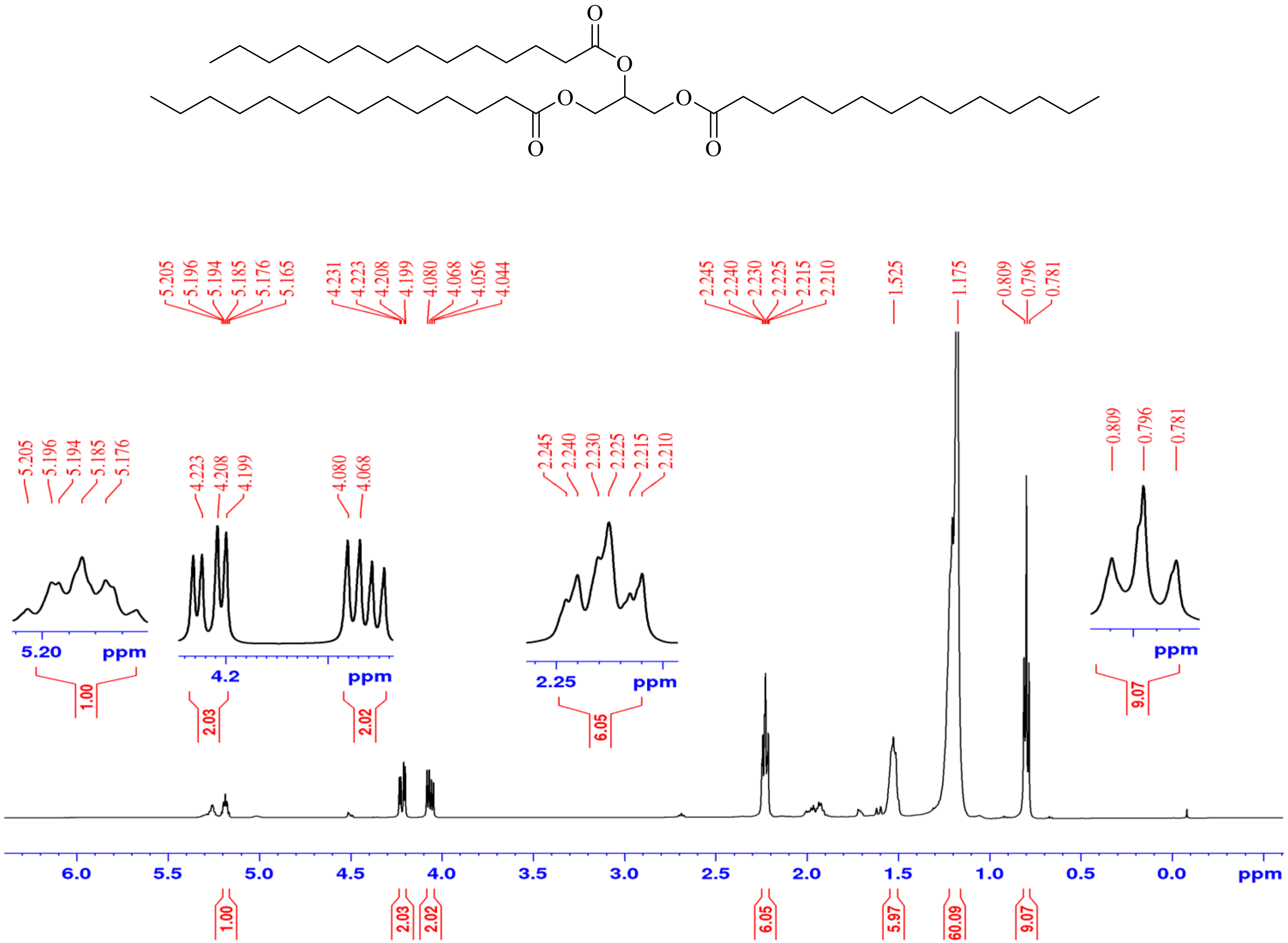

S17: ^{1}H NMR spectrum for compound **9** in $CDCl_3$ at 500 MHz

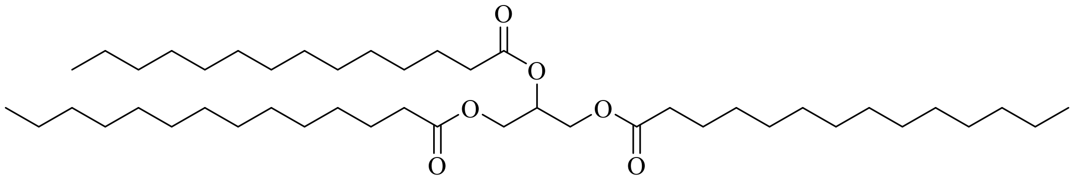

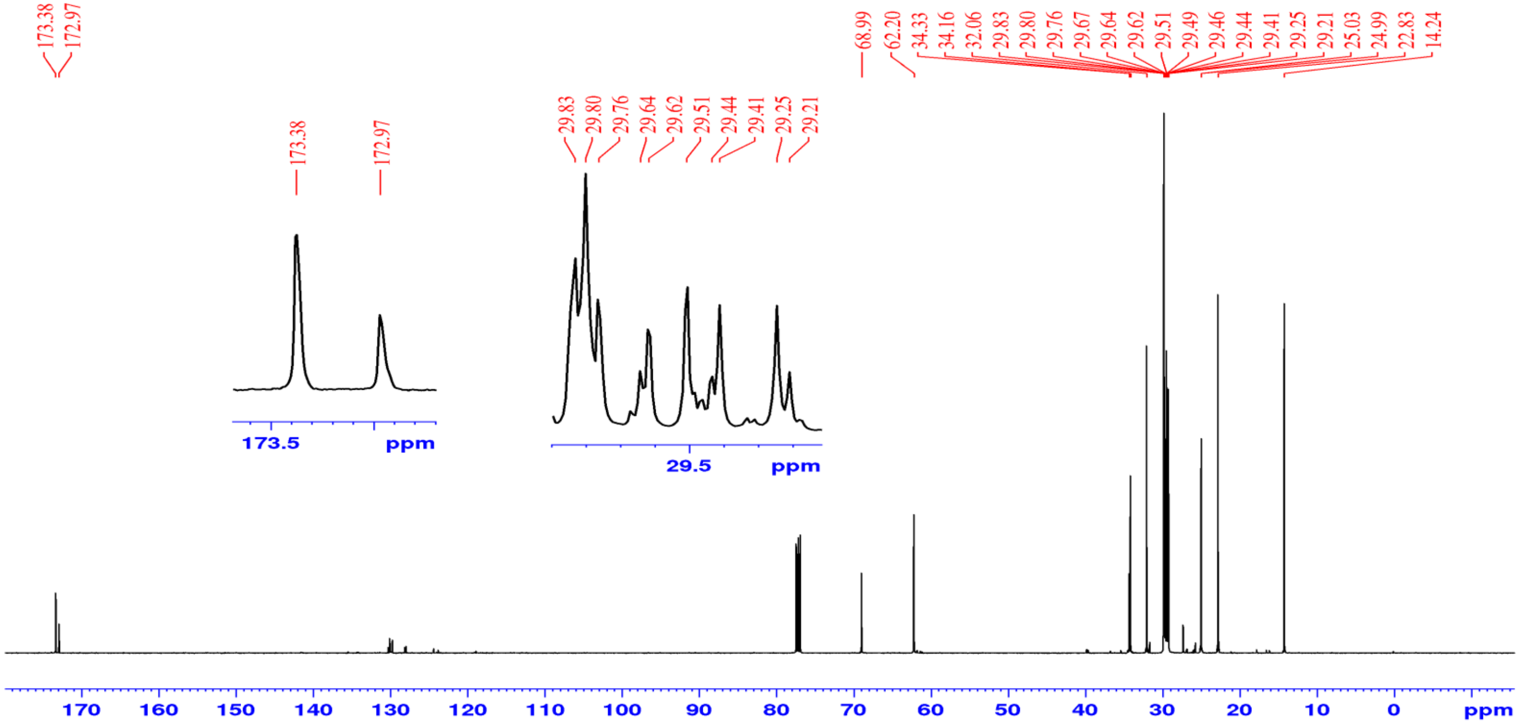

S18: ^{13}C NMR spectrum for compound **9**

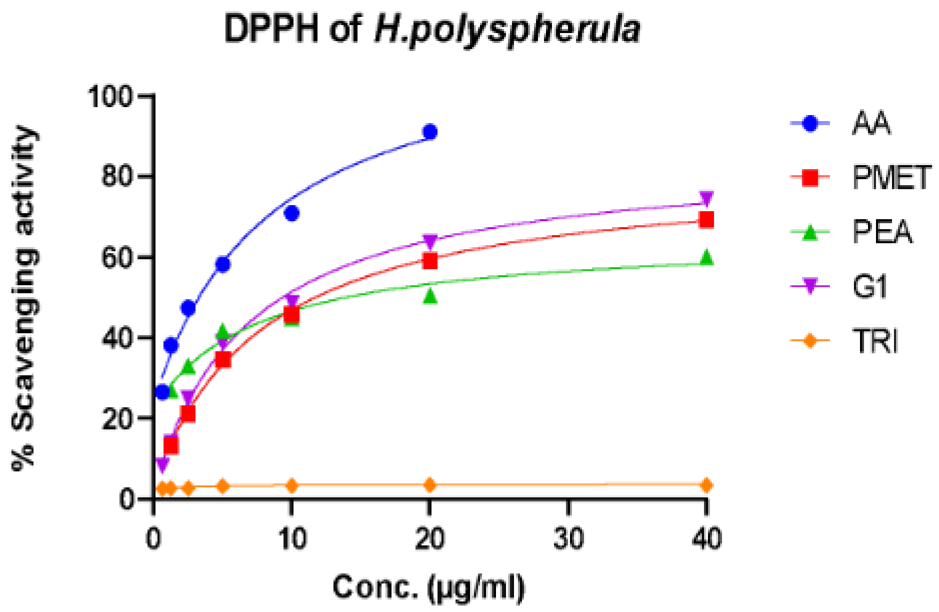

S19: Graphical resemblance of percentage scavenging activity between tested samples.

Legend: AA (ascorbic acid), PMET (methanol extract), PEA (ethyl acetate extract), G1 (3,4-dihydroxybenzoic acid), TRI (trimyristin)

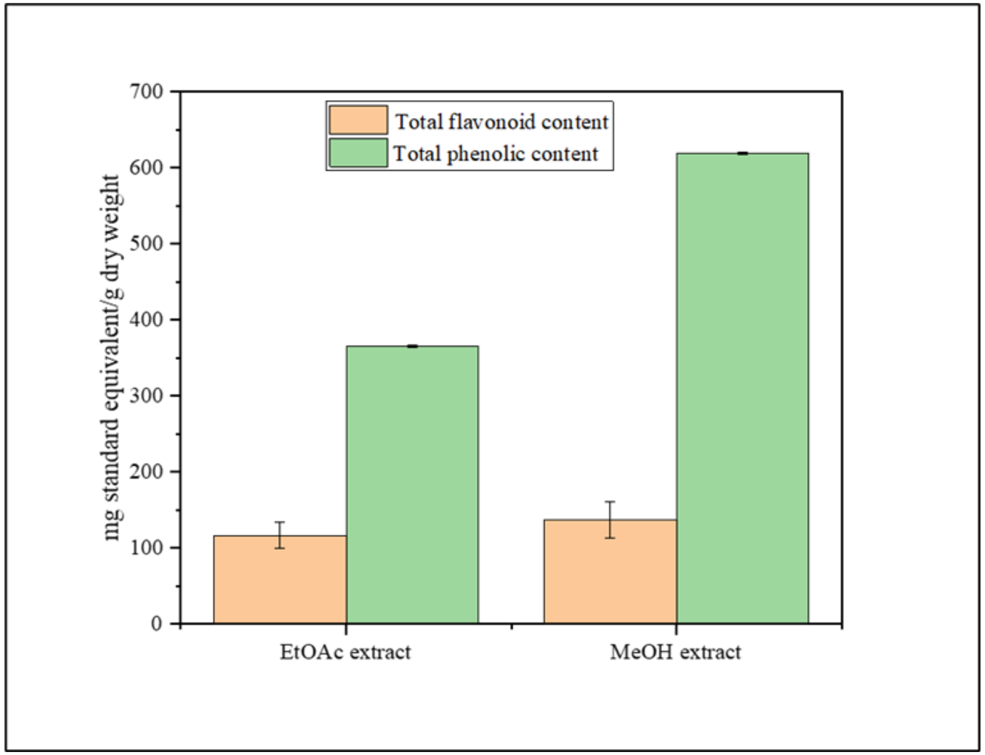

S20: Graphical presentation of total phenolic and total flavonoid contents of the extracts of *H.* polyspherula bark.

Values are expressed as mean ± standard deviation (n = 3).

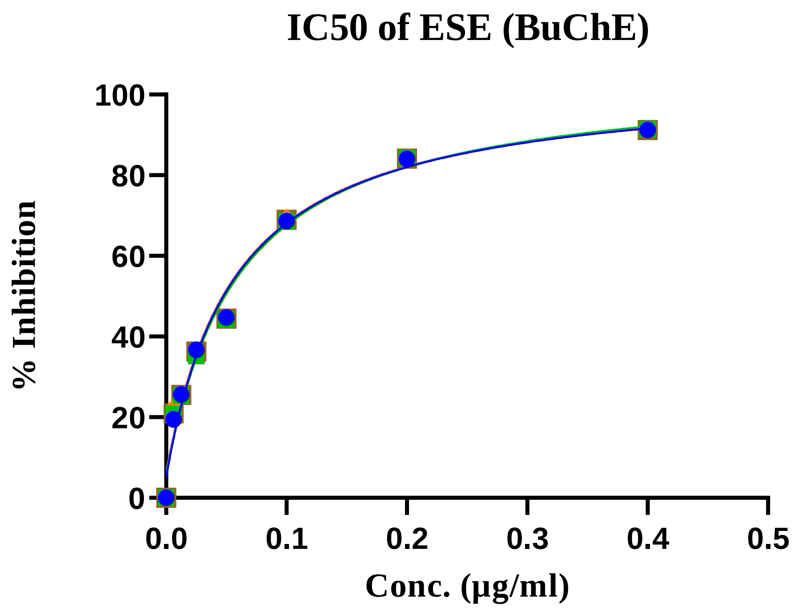

S21: Graphical presentation of IC_{50} *eserine* (positive control) against butyrylcholinesterase

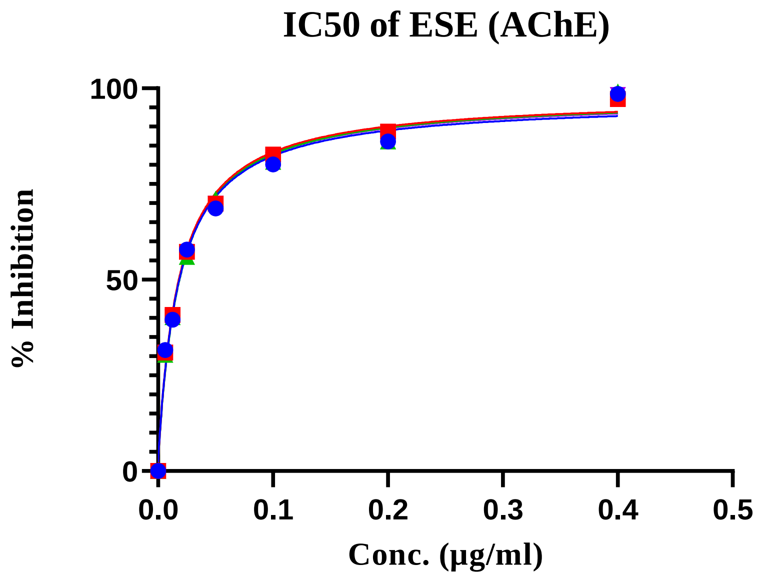

S22: Graphical presentation of IC_{50} *eserine* (positive control) against acetylcholinesterase

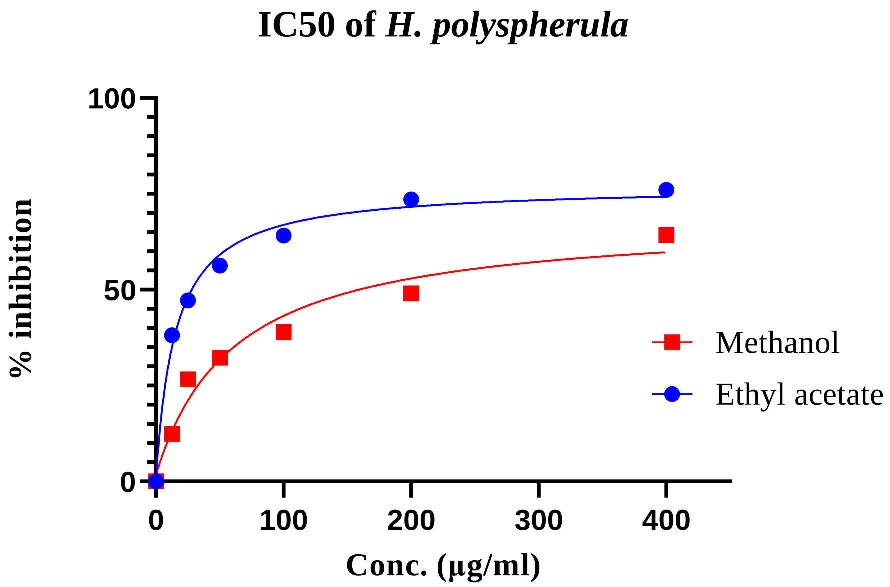

S23: Graphical relationship of IC_{50} of *H. polyspherula* extracts against butyrylcholinesterase.

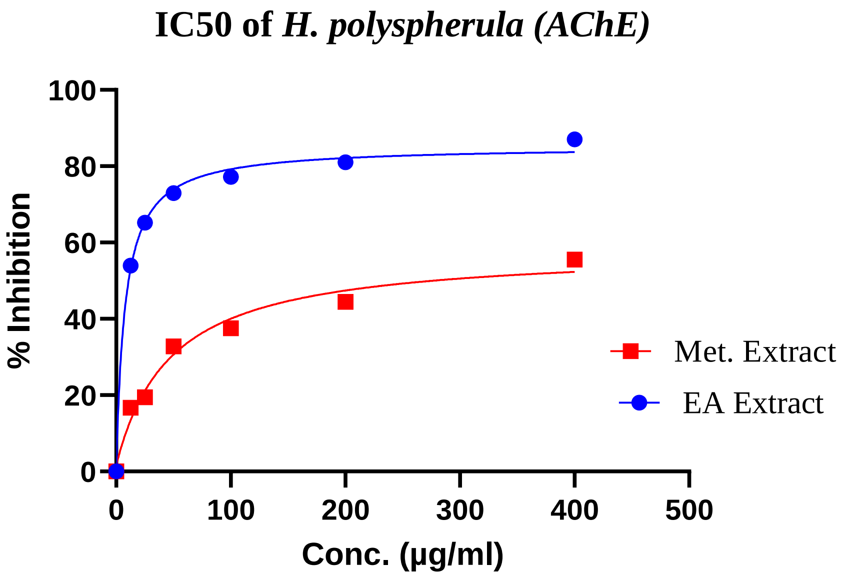

S24: Graphical relationship of IC_{50} of *H. polyspherula* extracts against Acetylcholinesterase.

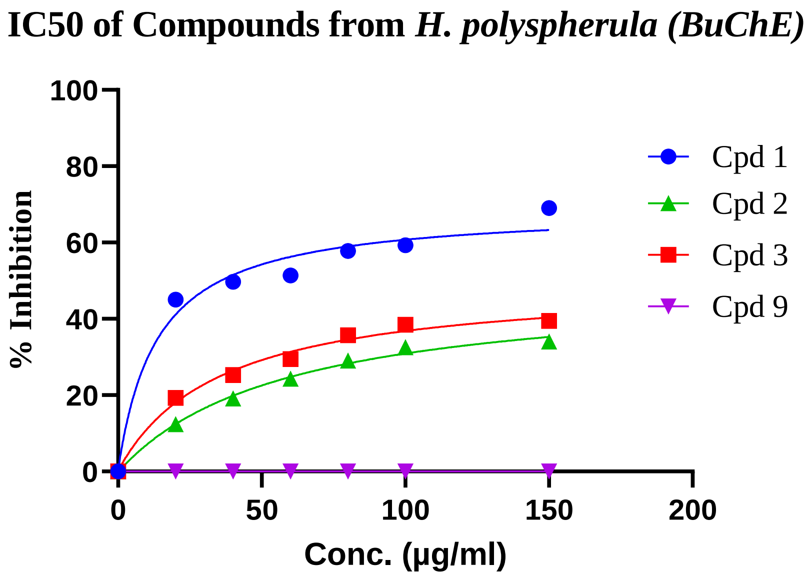

S25: Graphical relationship of IC_{50} of *H. polyspherula* compounds against Butyrylcholinesterase

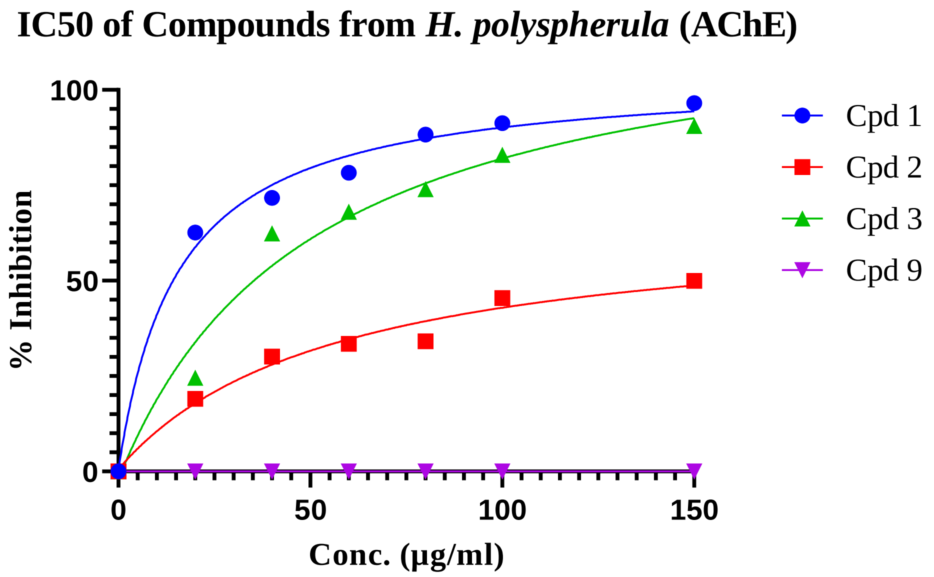

S26: Graphical relationship of IC_{50} of *H. polyspherula* compounds against Acetylcholinesterase
